# Lower Neighborhood Socioeconomic Status Associated with Reduced Diversity of the Colonic Microbiota in Healthy Adults

**DOI:** 10.1371/journal.pone.0148952

**Published:** 2016-02-09

**Authors:** Gregory E. Miller, Phillip A. Engen, Patrick M. Gillevet, Maliha Shaikh, Masoumeh Sikaroodi, Christopher B. Forsyth, Ece Mutlu, Ali Keshavarzian

**Affiliations:** 1 Department of Psychology and Institute for Policy Research, Northwestern University, Evanston, Illinois, United States of America; 2 Department of Internal Medicine, Division of Gastroenterology, Rush University Medical Center, Chicago, Illinois, United States of America; 3 Microbiome Analysis Center, Department of Environmental Science and Policy, George Mason University, Science and Technology Campus, Manassas, VA, United States of America; Loyola University Chicago, UNITED STATES

## Abstract

In the United States, there are persistent and widening socioeconomic gaps in morbidity and mortality from chronic diseases. Although most disparities research focuses on person-level socioeconomic-status, mounting evidence suggest that chronic diseases also pattern by the demographic characteristics of neighborhoods. Yet the biological mechanisms underlying these associations are poorly understood. There is increasing recognition that chronic diseases share common pathogenic features, some of which involve alterations in the composition, diversity, and functioning of the gut microbiota. This study examined whether socioeconomic-status was associated with alpha-diversity of the colonic microbiota. Forty-four healthy adults underwent un-prepped sigmoidoscopy, during which mucosal biopsies and fecal samples were collected. Subjects’ zip codes were geocoded, and census data was used to form a composite indicator of neighborhood socioeconomic-status, reflecting household income, educational attainment, employment status, and home value. In unadjusted analyses, neighborhood socioeconomic-status explained 12–18 percent of the variability in alpha-diversity of colonic microbiota. The direction of these associations was positive, meaning that as neighborhood socioeconomic-status increased, so did alpha-diversity of both the colonic sigmoid mucosa and fecal microbiota. The strength of these associations persisted when models were expanded to include covariates reflecting potential demographic (age, gender, race/ethnicity) and lifestyle (adiposity, alcohol use, smoking) confounds. In these models neighborhood socioeconomic-status continued to explain 11–22 percent of the variability in diversity indicators. Further analyses suggested these patterns reflected socioeconomic variations in evenness, but not richness, of microbial communities residing in the sigmoid. We also found indications that residence in neighborhoods of higher socioeconomic-status was associated with a greater abundance of *Bacteroides* and a lower abundance of *Prevotella*, suggesting that diet potentially underlies differences in microbiota composition. These findings suggest the presence of socioeconomic variations in colonic microbiota diversity. Future research should explore whether these variations contribute to disparities in chronic disease outcomes.

## Introduction

Chronic diseases pose a substantial economic and personal burden in developed countries across the world [1]. Socioeconomic disparities in morbidity and mortality from chronic disease have long been recognized [2–4]. Although much of this research has focused on person-level indicators of socioeconomic-status (SES), there is mounting evidence to suggest that chronic diseases also are patterned by the demographic characteristics of neighborhoods and communities. In prospective studies, residents of low-SES neighborhoods show higher rates of asthma, diabetes, myocardial infarction, stroke, functional limitations, and overall mortality in comparison to individuals from more affluent neighborhoods [5–9]. These associations often, though not always, remain significant following adjustment for person-level SES indicators, suggesting a direct influence of neighborhood characteristics on morbidity and mortality from these conditions.

There is increasing recognition that many chronic diseases share common pathologic features, in particular low-grade inflammation [10]. Many of these "inflammatory” conditions are also characterized by alterations in the composition of the intestinal microbiota [11–14]. The precise role of these commensal microorganisms in chronic disease pathogenesis remains unclear. However, preclinical studies demonstrate that gut microbiota plays a key role in food digestion and energy harvest, and in the process generate short-chain fatty acids (SCFA) and other metabolic products that regulate fat accumulation, gluconeogenesis, insulin sensitivity, and inflammation [15,16]. In clinical studies of various chronic health problems–including obesity, diabetes, inflammatory bowel disease, asthma, heart disease, and some cancers–a common though not universal finding is that affected subjects have a less diverse repertoire of gut microbes than healthy controls [17–21].

To our knowledge, the relationship between neighborhood SES and microbiota diversity has not yet been investigated. Nevertheless, there are many reasons to hypothesize that such an association would exist [22]. Indeed, many of the lifestyle and ecological characteristics shown to reduce gut microbial diversity are disproportionately prevalent in low-SES neighborhoods [23–29]. These factors include highly processed foods, physical inactivity, visceral adiposity, psychosocial stress, heavy antibiotic use, and exposure to pollutants, toxicants, and endocrine disruptors [16,30]. Accordingly, we conducted secondary analyses of an existing study of healthy control (HC) subjects, in which we had quantified the alpha-diversity of the bacterial microbial communities in both mucosal biopsy and fecal samples collected from the sigmoid colon. By geo-coding the subject’s zip codes, and linking them to publicly available databases from the census, we developed indicators of neighborhood SES, and examined their association with indices of colonic bacterial composition. We also examined whether the subjects’ demographic and lifestyle related characteristics might underlie these associations.

## Material and Methods

### Subjects

Healthy control (HC) volunteers (*n* = 44) were recruited via flyers and research advertisements at Rush University Medical Center (RUMC) as part of studies examining the relationship of alcohol intake and the intestinal microbiota (National Institute of Health (NIH)- National Institute on Alcohol Abuse and Alcoholism (NIAAA) grants: AA013745 and AA019405) [31–33]. In addition to the original studies, all HC subjects gave written informed consent to the use of their samples and data becoming part of an RUMC Institutional Review Board (IRB)-approved gastrointestinal (GI) repository. The research studies performed for this investigation was approved by the IRBs of Northwestern University and RUMC (ORA 14040404-IRB02). All subject characteristics are shown in Table 1.

**Table 1:** Characteristics of healthy control subjects.

Healthy Control Subjects (*n* = 44)	Mean (SD) or Percent	Range
Age (years)	39.1 (14.2)	20–72
Gender (Female)	61.4%	-
Caucasian	50.0%	-
African-American	34.1%	-
Body Mass Index (kg/m^2^)	27.9 (6.7)	19.6–45.4
Current Smoker	14.3%	-
Alcohol Use (years)	12.1 (9.9)	0–43
Median Household Income (2012 dollars)	58,042 (22,400)	20,100–129,570
Median Home Value (2012 dollars)	297,495 (123,762)	121,259–565,975
Percent Employed	89.1 (5.4)	65.3–95.6
Percent High School Graduates	86.4 (9.3)	61.8–99.5
Neighborhood SES Composite	0.05 (0.89)	-2.0 –+1.9
Sigmoid Mucosa Alpha-Diversity (Shannon)	6.3 (0.8)	4.3–7.7
Sigmoid Mucosa Alpha-Diversity (Chao1)	1009 (613)	124–2426
Feces Alpha-Diversity (Shannon)	7.8 (0.7)	5.7–8.8
Feces Alpha-Diversity (Chao1)	2841 (1175)	459–6182

HC subject inclusion and exclusion criteria are as follows: Inclusion criteria: (1) no gastrointestinal complaints, symptoms, or documented gastrointestinal chronic disease; (2) normal physical exam, complete blood count (CBC), and comprehensive metabolic profile; (3) consumption of no more than a moderate amount of alcohol {NIAAA definition [34]}; (4) no antibiotic, probiotic, and prescription medication use for at least three months; nor NSAID or high dose aspirin use for at least four weeks prior to sample collection. Low dose (81 mg/day) aspirin was allowed.

Exclusion criteria for HC subjects: (1) daily alcoholic beverage consumption; (2) at risk alcohol drinkers based on NIAAA criteria {*i*.*e*., females more than three drinks per day on a regular basis; for men more than four drinks per day} [35]; (3) unreliable drinking history; (4) the use of probiotics, antibiotics, and medications within three months prior to sample collection, (5) primary gastrointestinal pathology, (6) low platelet count (<80k), uncorrectable prolonged prothrombin time (>15 sec), or history of bleeding that precludes biopsies.

All HC subjects completed a RUMC GI research structured demographic and lifestyle questionnaires. All questionnaire packets were labeled by a sequential patient number to maintain patient confidentiality, and serve as the patient identifier for the study. HC subjects were not allowed to drink alcohol for 24h prior to the intestinal permeability test. All HC subjects lived in the Chicago-land area. For calculation of body mass index (BMI), each subject had height and weight measurements taken during their study visit at RUMC.

All work for this study was carried out in accordance with the Code of Ethics of the World Medical Association (Declaration of Helsinki) for experiments involving humans and Uniform Requirements for manuscripts submitted to biomedical journals published by the International Committee of Medical Journal Editors.

#### Neighborhood SES determinations

Neighborhood SES was derived from the subjects’ residential zip codes and publicly available datasets, collected as part of the 2010–2012 American Community Surveys. Each year, the US Census Bureau surveys approximately 3 million American households, sampled from all counties and equivalents. Data are pooled across survey years to obtain more reliable estimates, particularly in less densely populated areas. Using the online software Social Explorer (Oxford University Press, New York, NY USA), we obtained three-year estimates of each neighborhood’s median household income, educational (percent adults over 25 with high school diploma) and employment (percent adults over 25 seeking work) characteristics, and median owner-occupied home value. The indicators were strongly inter-correlated (*r*’s range: 0.61–0.75, *p* < 0.0005). We also ran an exploratory principal-component analysis (PCA), based on the matrix of inter-correlations between indicators. There was no factor rotation specified. The analysis yielded a single factor with an Eigen value = 3.09, which explained 77.18 percent of variance. No other factors with Eigen values > 0 emerged. Accordingly, we standardized each indicator, and then averaged them to form a neighborhood SES composite (Cronbach’s α = 0.90). Higher scores represented more affluent and educated neighborhoods.

#### Specimen collection

Mucosal biopsies (*n* = 41) and fecal samples (*n* = 26) were collected via endoscopy at the RUMC Endoscopy Lab. A limited, un-prepped sigmoidoscopy was performed using a standard adult upper endoscope (Olympus America Inc., Center Valley, PA USA) to 20–25 cm from the anal verge. Suction was not used during advancement of the scope and the biopsy forceps was not taken out of the channel of the scope until sample collection. Biopsies were taken from pink mucosa without visible feces at the sigmoid colon about 20 cm from the anal verge and were snap frozen in liquid nitrogen in the endoscopy room. An endoscopic luminal fecal sample was collected using a Roth Net (US Endoscopy Inc., Mentor, OH USA) from the lumen of the distal sigmoid colon during the procedure and also snap frozen in liquid nitrogen in the endoscopy room. Sigmoid mucosal biopsies and fecal samples were both stored at -80°C until the time of analysis. Endoscopic luminal feces were not collected for all HC subjects. Thus, this explains the differentiation between number values for feces compared to sigmoid mucosal biopsies.

#### Blood collection and serum isolation

Blood was collected via antecubital venipuncture into a BD (Becton, Dickinson and Company, NJ USA) Vacutainer Tube with clot activator and gel for serum separation, 10mL, red top (#367820) at the RUMC Endoscopy Lab. Serum tube was inverted five times upon collection, to ensure mixing of clot activator with blood. In an upright position, at room temperature, incubation blood clotting time was for 30 minutes. Whole blood was then centrifuged at 4.5x 1000 rpm for 15 minutes, with no brake, to separate blood from serum. At room temperature, serum was aspirated from blood tube, taking care not to disturb the cell layer or transfer any cells, aliquoted into cyrovials, and stored at -80°C until the time of analysis.

#### Microbiota profiling and bioinformatics analyses

Total DNA was extracted using a bead-beating commercially available kit, FastDNA Spin Kit for Soil, (MP Biomedicals, Solon, OH USA), using the manufacturer’s recommended protocol. Bacterial 16s rRNA sequencing was performed using our previously published Multitag Pyrosequencing (MTPS) protocol with universal forward primer 27F (5′- AGAGTTTGATCCTGGCTCA G-3′) and reverse primer 355R′ (5′-GCTGCCTCCCGTAGGAGT-3′) [36]. Briefly, a set of 96 forward primers that contain the 454 emulsion PCR linker-A and a different 8 base “barcode or tag” and 27F primer sequence were generated (Life Technologies, Carlsbad, CA USA). Also, the reverse primer (355R) was made with 454 linker-B attached and fluorescently labeled with FAM. We made the reverse primer labeled, to be able to fingerprint the PCR products for quality control [37]. A standard PCR was done with 32 cycles as described previously [38]. The samples ran on an ABI3130Xl capillary machine for fingerprinting, pooled based on the PCR product concentrations, purified using Ampure magnetic beads (Agencourt Biosciences, Beverly, MA USA), and quantitated with a DTX880 Multimode Fluorescent detector (Beckman Coulter, Pasadena, CA USA) for use in the emulsion PCR. The emulsion PCR was performed using 454 emPCR-LibL kit according to manufacturer’s protocol and processed further for sequencing on a GS Junior pyrosequencer (Roche, Switzerland). We used a custom PERL script to identify the barcode sequence in each read and associate the sequence reads with specific samples. The numbers of reads are presented in S1 Table. The taxa present within each sample were identified using the Bayesian analysis tool in version 10 of the Ribosomal Database Project (RDP10) [39]. The abundances of the bacterial identifications were then normalized using a custom PERL script and taxa present at >1% of the community were tabulated and the Shannon indices were calculated from the above abundance table using MVSP_v3.13 (Kovach, Wales, UK). Operational Taxonomic Units (OTUs) were also identified from the sequence data, in S2 Table, using the Quantitative Insights into Microbial Ecology (QIIME version 1.8.0) pipeline and the Chao1 index was calculated using the alpha-diversity script from the QIIME package [40]. The alpha-diversity indices and number of observed species per sample is presented in S3 Table. Histograms showing the overall microbiota composition at the Order and Family taxonomic levels, using greengenes database (release 13_8) in HC subject samples, are shown in S1, S2 and S3 Figs.[41] Alpha rarefaction plots are presented in S4 and S5 Figs. Raw sequence data (FASTQ files) were deposited in the NCBI Sequence Read Archive under project PRJNA287290. Canonical Correspondence analysis (MVSP_v3.13) was performed using the SES as the environmental variable and Family taxa level, which are presented in S6, S7 and S8 Figs.

#### Intestinal permeability and reagents

Intestinal permeability was determined by quantification of urinary excreted sugars after ingestion of an oral sugar cocktail [42–44]. The oral sugar cocktail was taken by the HC subjects seven days after the endoscopic sigmoid colon mucosa and fecal specimens were collected. Fasted HC subjects were given an oral cocktail of 40 g sucrose, 2 g mannitol, 7.5 g lactulose mixed in 8 oz. of water and 1 g sucralose in two capsules at 6 AM, and then urine was collected for 24 hours. Urinary concentrations of excreted sugars were determined using gas chromatography and an internal standard as previously described [42,44]. The intestinal permeability results were expressed as percent excretion of oral dose of the various sugars as previously described [42,44].

Lactulose (4-o-β-D-galactopyranosyl-D-fructofuranose) was obtained from Bertek Pharmaceuticals, as brand name Kristalose. Mannitol (D-mannitol) and sucrose (α-D-glucopyranosyl-β-D-fructofuranoside) were obtained from Sigma Aldrich. Sucralose (1,6-dichloro-1,6-dideoxy-β-D-fructofuranosyl-4-chloro-4-deoxy-α-D-glucopyranoside) was supplied by Tate and Lyle. Trifluoroacetic acid ammonia, sodium borodeuteride, acetone, acetic anhydride and glacial acetic acid were purchased at their highest grade purity from Sigma Aldrich.

#### Serum lipopolysaccharide

Serum lipopolysaccharide (LPS) was determined using the Lonza LPS quantitation kit HK315-01 (Hycult Biotech, The Netherlands) as described by the manufacturer [45].

#### Serum Cytokines

Serum levels of the cytokines interleukins- 6, 8, and 10, as well as tumor necrosis factor-α were determined using the MILLIPLEX® Multiplex Assays Using Luminex (EMD Millipore, Billerica, MA USA).

#### Statistical analyses

Statistical analyses were performed in IBM SPSS software, Version 22 (Armonk, NY USA). To check for departures from normality, histograms were generated for all predictors and outcomes, and skew and kurtosis statistics were computed. All of the diversity indices and major predictors conformed to normal distributions, so transformations were unnecessary. To clarify the relationship between neighborhood SES and microbiota diversity, we constructed a series of hierarchical linear regression equations. The first sets of models were unadjusted, and simply regressed the outcome of interest (one of the four microbiota diversity indicators) onto the neighborhood SES composite. In a subsequent series of models, we entered the four demographic covariates (age, gender, and self-identified racial/ethnic background, represented by dummy codes to reflect membership in Caucasian and African-American subgroups) as a block in Step 1, followed by the neighborhood SES composite in Step 2. The final series of analyses used a parallel strategy, but entered four lifestyle covariates in Step 1 (BMI, as well as self-report of current smoking status (no/yes) and duration of alcohol use (in years)), followed by the neighborhood SES composite in Step 2. For each model, we report unstandardized regression coefficients, standard errors and confidence intervals, along with standardized coefficients (to allow comparisons among model predictors), *p* values, and the increment in R^2^ when the neighborhood SES composite was added to the model (to provide an estimate of variance explained).

## Results

### Preliminary analyses

Table 1 presents the HC subject’s sample characteristics. As can be seen, the HCs were healthy, middle-aged adults, and diverse with regard to gender, racial background, neighborhood SES, and microbial diversity within specimen type. On each of the indicators that comprised the SES composite, the sample had a considerable amount of range (e.g., at the low end, median annual household income was $20,100 and unemployment rates were almost 35%, whereas at the high end, income was $129,570 and unemployment was under 5%). The two indicators of microbiota related alpha-diversity—Shannon index and Chao1 richness—were strongly correlated within specimen types. For sigmoid biopsies, the Shannon and Chao1 indices were correlated at *Spearman r* = 0.60, *p* < 0.0001; for the fecal specimens these indices were correlated at *Spearman r* = 0.68, *p* < 0.0002. However, each specimen type had its own distinct microbial composition, as reflected in the non-significant correlation between sigmoid biopsy and fecal specimen values for the Shannon (*Spearman r* = 0.18, *p* = 0.40), and Chao1 (*Spearman r* = 0.15, *p* = 0.50) indices. To summarize, the Shannon and Chao1 indices reveal similar patterns of diversity within the sample of sigmoid biopsies, and also within the sample fecal specimens. However, there is very little overlap across these specimen types; the diversity of sigmoid biopsies is not indicative of the diversity of fecal specimens, and vice versa.

#### Neighborhood SES and microbiota diversity

**Fig 1:**
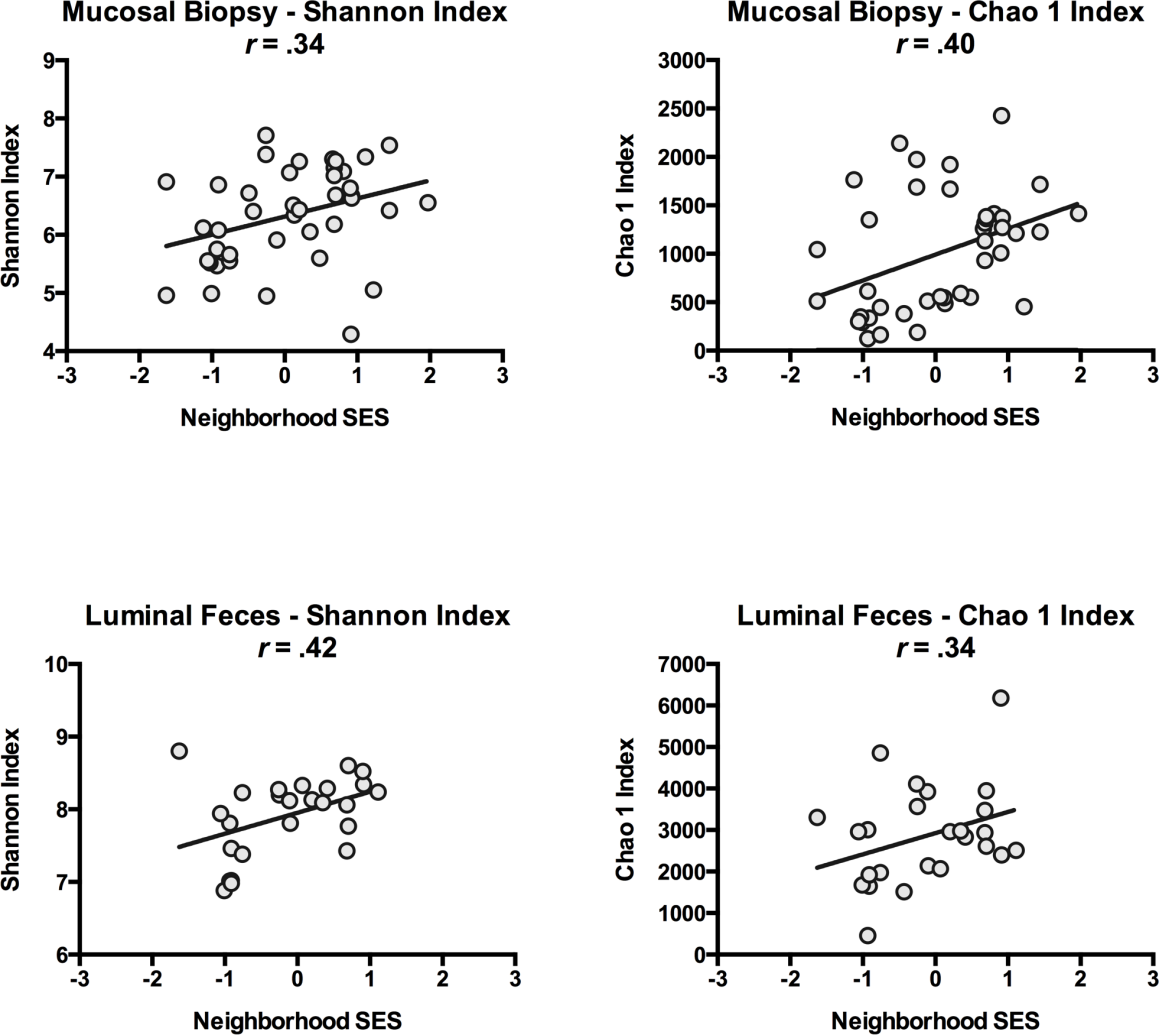
Scatter-plot of the neighborhood SES and alpha-diversity. Figs depict associations between neighborhood SES and alpha-diversity in biopsies excised from sigmoid mucosa (upper plots) and feces collected from sigmoid lumen (lower plots). Shannon and Chao1 indices are different metrics for calculating alpha-diversity.

**Fig 2:**
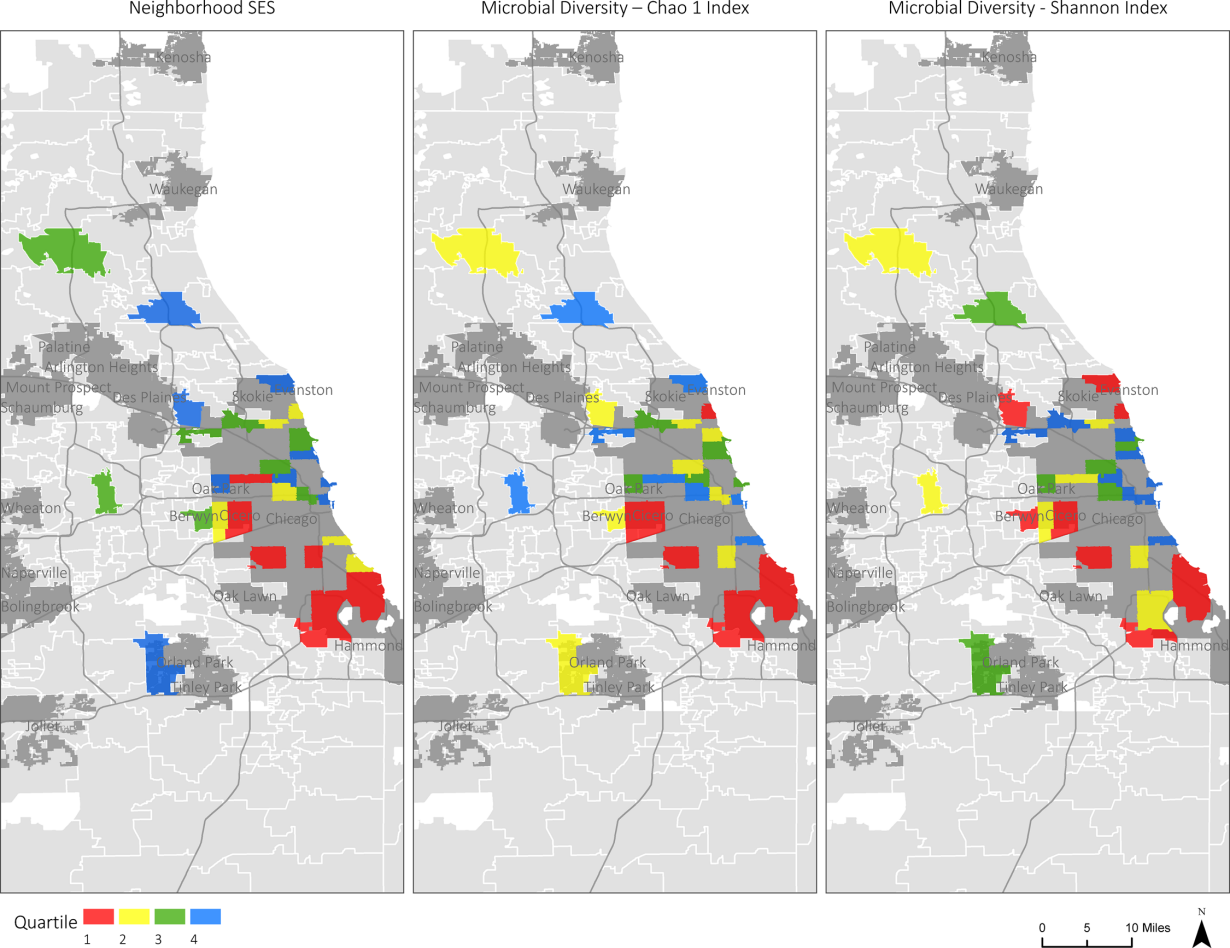
Map depicting the neighborhood SES and alpha-diversity. Map of greater Chicago region, illustrating associations between neighborhood SES and alpha-diversity in biopsies excised from sigmoid mucosa. Depicted are neighborhood SES (left panel), and two metrics of diversity, the Shannon (middle panel) and Chao1 (right panel) indices. For each variable, the sample was divided into quartiles, and neighborhoods were colored as follows: Red = lower 25% of sample; Yellow = 26–50%; Green = 51–75%; Blue = upper 25%. Geospatial data sources: Urban place area from TIGER/Line US Census 2010; Illinois and Wisconsin zip codes from TIGER/Line US Census 2010; Water area from TIGER/Line US Census 2010; Interstate lines from TIGER/Line US Census 2010.

Table 2 presents the results of linear regression analyses predicting microbiota diversity from the neighborhood SES composite. In these unadjusted analyses, neighborhood SES was positively associated with alpha-diversity indicators in both sigmoid biopsies and fecal specimens at the OTU level, and explained 12–18 percent of the sample-wide variability in these indicators. All of these associations were statistically significant (*p* < 0.04), with the exception of the Chao1 estimate of fecal specimen diversity (*p* = 0.09). It bears noting, however, that this association was of the same magnitude as others (*R*^*2*^ = 0.12), but with the smaller number of fecal specimens available (*n* = 25), it did not reach the significance threshold. Figs 1 and 2 illustrate these findings.

**Table 2:** Results of linear regression analyses predicting alpha-diversity indices from neighborhood SES.

	Sigmoid Mucosa Shannon Index *n* = 41	Sigmoid Mucosa Chao1 Index *n* = 41	Feces Shannon Index *n* = 26	Feces Chao1 Index *n* = 25
Unstandardized Coefficient (B)	0.31	0.27	0.29	0.51
Standard Error B	0.14	0.10	0.13	0.29
95% Confidence Interval	.03, .59	.07, .47	.02, .56	-.09, 1.12
Standardized Coefficient (β)	0.34	0.40	0.42	0.34
*P*-Value	0.03*	0.01*	0.04*	0.09
Variance Explained (R^2^)	0.12	0.16	0.18	0.12

*P-Value < 0.05

We performed follow-up analyses to determine whether these associations at the OTU level were independent of potential demographic and lifestyle characteristics. S4 Table presents correlations between these characteristics and neighborhood SES and microbiota related alpha-diversity. To the extent they lived in higher-SES neighborhoods, HC subjects were less likely to be African-American (*r* = -0.51, *p* < 0.0007) and had lower BMI (*r* = -0.39, *p* = 0.01). None of the characteristics was associated significantly with microbiota related alpha-diversity indices (*p* < 0.20). However, consistent with previous research [29,46–48], HC subjects with higher BMI values had less alpha-diversity across indices and specimens (*r*’s range: -0.12 to -0.27, *p*’s range: 0.09 to 0.44).

Table 3 shows the results of hierarchical regression analyses, where diversity indices at the OTU level were predicted from neighborhood SES, after adjusting for demographic (top panel) and lifestyle (bottom panel) characteristics. As can be seen, these factors had little effect on the strength of the associations between neighborhood SES and microbiota related alpha-diversity, with R^2^ values continuing to range from 11 to 18 percent. In two instances, the p values for these associations dropped below the conventional significance threshold (adjusted *p* = 0.08 and 0.09). But this was a consequence of covariates reducing model degrees of freedom, rather than any changes in the strength of the associations themselves (as reflected in the similarity of the R^2^ values between unadjusted and adjusted models).

**Table 3:** Results of adjusted linear regression analyses, predicting alpha-diversity indices from neighborhood SES, plus demographic and lifestyle covariates.

	Sigmoid Mucosa Shannon Index *n* = 41	Sigmoid Mucosa Chao1 Index *n* = 41	Feces Shannon Index *n* = 26	Feces Chao1 Index *n* = 25
**Adjusted for Demographic Covariates**				
Unstandardized Coefficient (B)	0.35	0.29	0.35	0.66
Standard Error B	0.17	0.12	0.16	0.36
Standardized Coefficient (β)	0.39	0.42	0.52	0.43
*P*-Value	0.09	0.02*	0.04*	0.08
Incremental Variance (ΔR^2^)	0.11	0.13	0.18	0.13
**Adjusted for Lifestyle Covariates**				
Unstandardized Coefficient (B)	0.45	0.30	0.28	0.77
Standard Error B	0.18	0.13	0.16	0.30
Standardized Coefficient (β)	0.45	0.41	0.42	0.54
*P*-Value	0.02*	0.03*	0.09	0.02*
Incremental Variance (ΔR^2^)	0.14	0.12	0.13	0.22

*P-Value < 0.05

Values reflect association of neighborhood SES with alpha-diversity indicators. Demographic covariates include age, gender, and dummy codes for Caucasian and African-American. Lifestyle covariates include body mass index, smoking status, and alcohol use.

One of the microbiota related alpha-diversity indicators, the Shannon index used above, is a composite measure, where higher values can reflect both richness (a larger number of distinct microbial taxa) and evenness (suggesting comparable relative abundances of the taxa without dominance of one or more of them within the sample). To clarify the link between SES and microbiota related alpha-diversity, we conducted secondary analyses exploring indices of richness and evenness within specific taxa. Because the distribution of these indices was highly skewed, and resisted efforts at transformation, we used Spearman rank-order correlations to test for microbiota associations. In the sigmoid biopsies, higher neighborhood SES was associated with greater evenness at the phylum level (*r* = 0.36, *p* = 0.02), but not the family or genus levels (*r* < 0.13, *p* > 0.42, *n* = 41). Higher neighborhood SES was not associated with richness at any of these levels (*r* < 0.26, *p* > 0.10, *n* = 41). In the fecal specimens, there were no associations between neighborhood SES and richness and evenness at any taxonomic level (*r* < 0.24, *p* > 0.22, *n* = 26).

#### Neighborhood SES and Enterotypes

**Fig 3:**
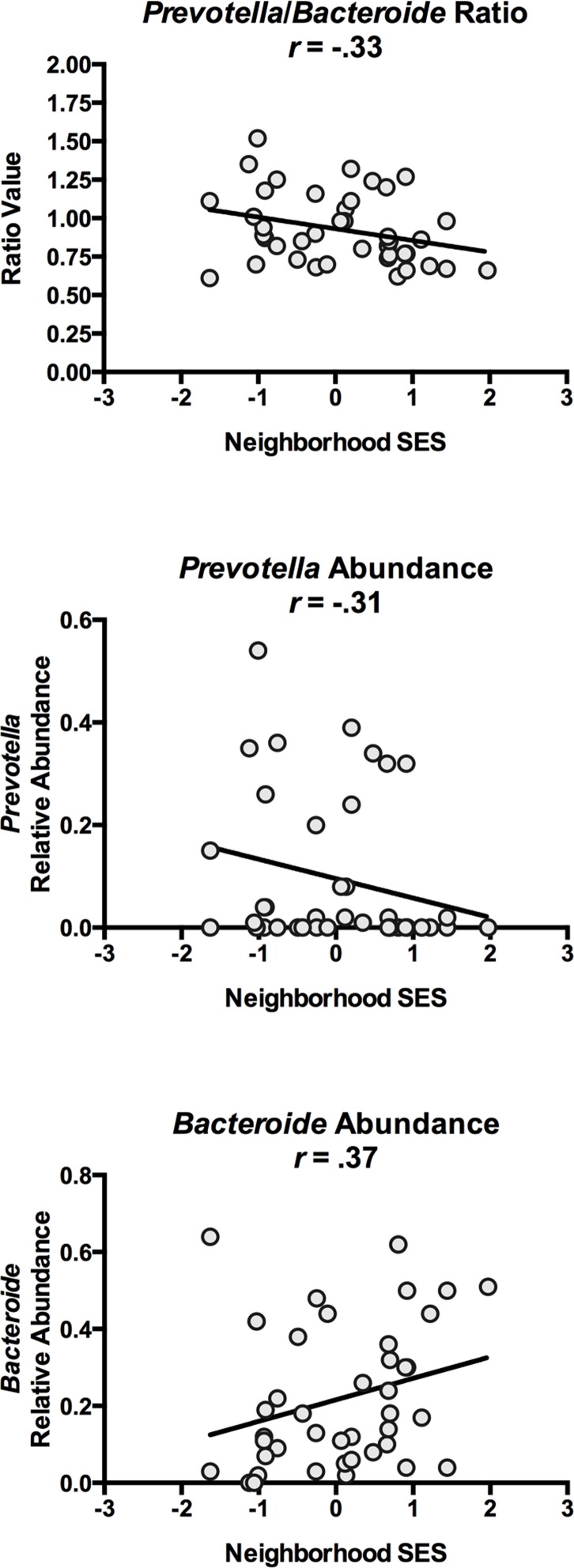
Scatter-plot of neighborhood SES and bacterial genera. Figs depict associations between neighborhood SES and *Prevotella* to *Bacteroides* ratio (upper panel), as well as relative abundance of *Prevotella* (middle panel) and *Bacteroides* (lower panel). Specimens are biopsies excised from sigmoid mucosa.

We also examined whether SES relates to the presence of specific microbial communities that have been implicated in chronic diseases of aging [49–55]. The focus here was on the relative abundance of genera sometimes used to categorize humans into enterotypes. These genera include *Bacteroides*, *Prevotella*, and *Ruminococcus*, whose abundances may reflect diets relatively enriched for animal fats, carbohydrates, and alcohol, respectively [16,32,48,56]. Because these variables were not distributed normally, we estimate their association with neighborhood SES using Spearman rank-order correlations, which are robust to violations of normality. As Table 4 shows, to the extent they resided in higher SES neighborhoods, HC subjects displayed a greater abundance of *Bacteroides* and a lower abundance of *Prevotella* in their sigmoid biopsies (*p*’s = 0.05 and 0.02, respectively). Higher neighborhood SES was also associated with a smaller ratio of *Prevotella* to *Bacteroides* (*p* = 0.04). These findings are illustrated in Fig 3. Neighborhood SES was unrelated to mucosal *Ruminococcus* abundance (*p* = 0.77). We were unable to perform similar analyses in the fecal specimens because of a lack of variability in the abundance estimates. (22/26 HC subjects had no detectable fecal *Prevotella*, and 14/26 had no detectable fecal *Bacteroides)*.

**Table 4:** Spearman rank-order correlations between neighborhood SES and bacterial genera reflecting sigmoid mucosal enterotypes.

	*Prevotella n* = 41	*Bacteroides n* = 41	*P/B* Ratio *n* = 41	*Ruminococcus n* = 41
Rank-order correlation	-0.31	0.37	-0.33	-0.05
*P*-Value	0.05*	0.02*	0.04*	0.77
Variance Explained (R^2^)	0.10	0.14	0.11	0.03

*P-Value < 0.05

*P/B* = *Prevotella* to *Bacteroides* Ratio

We also tested whether neighborhood SES related to the abundance of *Firmicutes* or *Bacteriodetes*, the two predominant phyla in the gut, which are centrally involved in energy harvest and metabolism. However, these associations were non-significant (*r* < 0.10, *p* > 0.53).

#### Markers of gut leakiness to endotoxin and systemic inflammation

In addition to gut-microbiota composition, changes in the intestinal barrier function can also contribute to gut-derived systemic inflammation [57–60]. Furthermore, a cross-talk between intestinal microbiota and intestinal mucosa has been demonstrated suggesting that changes in microbiota composition can impact intestinal barrier function (and vice versa) [61–63]. Accordingly, we measured serum endotoxin (*e*.*g*., LPS) content, as well as urinary excretion of lactulose and sucralose after oral challenge with these sugars (5 and 24 hours later, respectively). Neighborhood SES was not associated with any of these markers of gut permeability, however (*R*^*2*^ values < 0.01, *p* > 0.55).

Gut microbiota are known to regulate inflammation [12,22], a pathologic feature of many chronic diseases [10]. We therefore measured serum levels of the cytokines interleukins-6, 8, and 10, as well as tumor necrosis factor-α. However, neighborhood SES was unrelated to the levels of these cytokines, (*R*^*2*^ values < 0.04, *p* > 0.30).

## Discussion

In the United States, there are persistent and widening socioeconomic gaps in morbidity and mortality from chronic diseases, including asthma, diabetes, coronary heart disease, and some cancers [2,3,64–67]. There is increasing recognition that chronic diseases share common pathogenic features, some of which may involve alterations in the composition, diversity, and functioning of the gut microbiota [11–14]. Accordingly, we examined whether neighborhood SES was associated with microbiota related alpha-diversity in a sample of healthy adults. We observed significant relationships across two distinct microbiota communities, residing in the mucosal and luminal regions of the sigmoid colon, and these associations were consistent across two distinct indicators of alpha-diversity. The direction of these associations was positive, meaning that as neighborhood SES increased, so did the level of microbiota related alpha-diversity. Further analyses suggested these patterns reflected socioeconomic variations in the evenness, but not the richness, of microbial communities residing in the sigmoid, and primarily on the mucosal surface. In other words, as neighborhood SES increased, HC subjects displayed more even distributions of intestinal microbial populations. Furthermore, we found preliminary indications that residence in higher SES neighborhoods is associated with a greater abundance of *Bacteroides* and a lower abundance of *Prevotella*. To our knowledge, this is the first report of socioeconomic disparities in microbiota composition.

Because of the study’s observational cross sectional design, we cannot make cause-and-effect inferences about these microbiota associations. The adjusted regression analyses suggest that neighborhood SES is not simply acting as a proxy for other demographic characteristics, like age, gender, or racial/ethnic background, which are known potential correlates of gut microbiota composition [51]. We also did not find evidence that SES’s relationship with alpha-diversity was mediated through lifestyle factors that included adiposity, smoking, or alcohol consumption. With that said, future research will need more extensive measures of lifestyle to fully understand these relationships, with a particular focus on factors we did not measure here, like visceral fat, physical activity, dietary intake, and frequency of antibiotic use, especially in childhood.

With regard to the dietary habits, our findings suggest the hypothesis that at least in the greater Chicago area, individuals living in more affluent neighborhoods might have diets that are enriched for animal products relative to carbohydrates, possibly accounting for the alterations seen in the abundances of *Bacteroides* and *Prevotella* [56,68]. Subsequent research with in-depth dietary monitoring is needed to verify and clarify the interpretation of these results.

Two potential alternative explanations for our findings should also be considered. First, it is unclear to what degree the observed associations reflect neighborhood vs. household SES. In many previous studies, distinct neighborhood influences on health have been observed [5–9]. Nevertheless, it is certainly plausible that our composite is acting, to some degree, as a proxy for material resources, educational attainment, or other socioeconomic characteristics of subjects’ households. We cannot evaluate this possibility here, because the data comes from a larger study, which had a limited panel of demographic indicators. In future research, it will be important to consider household and neighborhood SES in parallel, and determine their relative associations with microbiota composition. It also will be informative to disaggregate the composite to determine if specific components of neighborhood SES are more or less strongly related to microbiota outcomes. Because of the small sample and associated risk of Type 1 error, we did not perform such analyses here. Second, it is possible that the associations found here reflect a causal influence of the microbiota itself, rather than SES. In mice, the intestinal microbiota plays a role in brain development and behavioral regulation across the life course [69]. Although it remains unclear whether similar dynamics operate in humans, the mouse findings raise the possibility of a reverse-directionality scenario, wherein microbiota composition affects socioeconomic attainment, and consequently the resources a person has available for housing in specific neighborhoods. Prospective research is needed to address this possibility.

Despite these limitations and uncertainties, the findings reported here are innovative in documenting a relationship between neighborhood SES and microbiota composition and alpha-diversity, which is evident in both mucosal and luminal microbiota communities of the sigmoid colon. The associations we observed, although statistically significant, were generally modest in size, with neighborhood SES explaining up to 10–22% of the person-to-person variation in diversity indices. Clearly, factors other than neighborhood SES contribute to microbiota composition. With that said, the degree of variation explained by neighborhood SES exceeded that of person-level factors often thought to be important determinants of microbiota composition, including age, race/ethnicity, and BMI (see Table 2 and S5 Table). Thus, if substantiated in future research, these findings may help clarify the mechanistic basis of socioeconomic disparities in chronic disease, and may offer insights into preventive or therapeutic interventions that could ameliorate these disparities.

## Supporting Information

S1 Table Numbers of reads per endoscopic specimen sample. The numbers of sequence reads per healthy control subject’s endoscopic specimen sample. Healthy control subjects (N = 44). A total of N = 67 samples: N = 41 sigmoid, N = 26 feces.
(DOCX)

S2 Table Summary of the number of OTUs across healthy control samples at each taxonomic levels. The number of OTUs calculated from RDP10 tables for each taxonomic resolution (Phylum to Genus) with QIIME analysis. Healthy control subjects (N = 44). A total of N = 67 samples: N = 41 sigmoid, N = 26 feces.
(DOCX)

S3 Table Alpha-diversity statistics and observed species number from QIIME's alpha-diversity.py. The alpha-diversity statistics and observed species numbers from QIIME sequence clusters. Healthy control subjects (N = 44). A total of N = 67 samples: N = 41 sigmoid, N = 26 feces.
(DOCX)

S4 Table Correlations between neighborhoods SES, alpha-diversity indices, and covariates. Values are Pearson (for age, body mass index, alcohol) or Point-Biserial correlations (for Gender, Caucasian, African-American, and Smoking). Gender is coded as 0 = Male, 1 = Female. Caucasian, African-American, and Smoker are coded as 0 = No, 1 = Yes. For sigmoid mucosa, where *n* = 41, the critical value of *r* at α = 0.05 is 0.30. For Feces, where *n* = 26, the critical value of *r* at α = 0.05 is 0.37. * *p* < 0.01; ^^^ *p* < 0.001.
(DOCX)

S5 Table Percent variance in alpha diversity explained by covariates. Table shows R^2^ values, reflecting percentage of variance in alpha diversity indices explained by each covariate (for age, body mass index, alcohol). Gender is coded as (0 = Male), (1 = Female). Caucasian, African-American, and Smoker are coded as (0 = No), (1 = Yes).
(DOCX)

S1 Fig Histogram of Order abundances sorted by SES value. The QIIME taxa dominant abundances are presented as stacked histograms. Healthy control endoscopic specimen samples were sorted by the SES values from positive (left side of graph) to negative (right side of graph). The Bacteroidales is highlighted in blue.
(TIF)

S2 Fig Histogram of Family abundances sorted by SES value. The QIIME taxa dominant abundances are presented as stacked histograms. Healthy control endoscopic specimen samples were sorted by the SES values from positive (left side of graph) to negative (right side of graph). The Bacteroidaceae is highlighted in blue.
(TIF)

S3 Fig Histogram of Family emphasized abundances sorted by SES value. The QIIME taxa abundances are presented as stacked histograms. Healthy control endoscopic specimen samples were sorted by the SES values from positive (left side of graph) to negative (right side of graph). The Bacteroidaceae (blue), Prevotellaceae (green), and Ruminococcaceae (yellow) are highlighted.
(TIF)

S4 Fig Endoscopic specimen Alpha Rarefaction Observed Species. Sample rarefaction using Alpha_Diversity.py was run on the data and the sequences per healthy control endoscopic specimen sample was plotted. The rarefaction curves are done with OTUs from QIIME. The rarefaction analysis indicates that the community for most of the endoscopic specimen samples was close to being saturated.
(TIF)

S5 Fig Endoscopic specimen Alpha Rarefaction Chao1. Sample rarefaction using Alpha_Diversity.py was run on the data and the sequences per healthy control endoscopic specimen sample was plotted. The rarefaction curves are done with OTUs from QIIME. The rarefaction analysis indicates that the community for most of the endoscopic specimen samples was close to being saturated.
(TIF)

S6 Fig Endoscopic specimen sample Canonical Correspondence analysis. Canonical Correspondence Analysis (CCA) plot of all healthy control endoscopic specimen samples using SES as the environmental variable. SES composite CCA case scores indicated on X-axis (low SES is left; high SES high is right).
(TIF)

S7 Fig Endoscopic specimen Family level taxa Canonical Correspondence analysis. Canonical Correspondence Analysis (CCA) plot of all healthy control endoscopic specimens Family level taxa using SES as the environmental variable. SES composite CCA case scores indicated on X-axis (low SES is left; high SES high is right).
(TIF)

S8 Fig Endoscopic specimen combined Canonical Correspondence analysis. Canonical Correspondence Analysis (CCA) bi-plot of all healthy control endoscopic specimen samples and Family level taxa using SES as the environmental variable. SES composite CCA case scores indicated on X-axis (low SES is left; high SES high is right).
(TIF)
