# Supplementary material for: Lower Neighborhood Socioeconomic Status Associated with Reduced Diversity of the Colonic Microbiota in Healthy Adults
Source: PLoS One. 2016 Feb 9;11(2):e0148952. doi: 10.1371/journal.pone.0148952 (PMC4747579; doi:10.1371/journal.pone.0148952)
Supplement: S1 Table — The numbers of sequence reads per healthy control subject’s endoscopic specimen sample. Healthy control subjects (N = 44). A total of N = 67 samples: N = 41 sigmoid, N = 26 feces. (DOCX) [file pone.0148952.s001.docx]

**S1 Table. Numbers of reads per healthy control subject’s sample.**

| **Study Codes** | **Endoscopic Sample Type** | **Reads** |
| --- | --- | --- |
| Healthy Control 1 | Sigmoid Mucosa | 1629 |
| Healthy Control 2 | Sigmoid Mucosa | 1588 |
| Healthy Control 3 | Sigmoid Mucosa | 2023 |
| Healthy Control 4 | Sigmoid Mucosa | 2482 |
| Healthy Control 5 | Sigmoid Mucosa | 1305 |
| Healthy Control 6 | Sigmoid Mucosa | 1184 |
| Healthy Control 7 | Sigmoid Mucosa | 3371 |
| Healthy Control 8 | Sigmoid Mucosa | 1037 |
| Healthy Control 9 | Sigmoid Mucosa | 4276 |
| Healthy Control 10 | Sigmoid Mucosa | 1269 |
| Healthy Control 11 | Sigmoid Mucosa | 1187 |
| Healthy Control 12 | Sigmoid Mucosa | 1805 |
| Healthy Control 13 | Sigmoid Mucosa | 1762 |
| Healthy Control 14 | Sigmoid Mucosa | 1720 |
| Healthy Control 15 | Sigmoid Mucosa | 1753 |
| Healthy Control 16 | Feces | 2850 |
| Healthy Control 17 | Feces | 1908 |
| Healthy Control 17 | Sigmoid Mucosa | 2070 |
| Healthy Control 18 | Feces | 2296 |
| Healthy Control 18 | Sigmoid Mucosa | 2539 |
| Healthy Control 19 | Feces | 2194 |
| Healthy Control 20 | Sigmoid Mucosa | 1251 |
| Healthy Control 20 | Feces | 2329 |
| Healthy Control 21 | Sigmoid Mucosa | 233 |
| Healthy Control 21 | Feces | 2749 |
| Healthy Control 22 | Feces | 729 |
| Healthy Control 22 | Sigmoid Mucosa | 2492 |
| Healthy Control 23 | Sigmoid Mucosa | 1256 |
| Healthy Control 23 | Feces | 2715 |
| Healthy Control 24 | Feces | 1548 |
| Healthy Control 24 | Sigmoid Mucosa | 9282 |
| Healthy Control 25 | Sigmoid Mucosa | 1174 |
| Healthy Control 25 | Feces | 2812 |
| Healthy Control 26 | Sigmoid Mucosa | 222 |
| Healthy Control 26 | Feces | 2555 |
| Healthy Control 27 | Feces | 2282 |
| Healthy Control 28 | Sigmoid Mucosa | 527 |
| Healthy Control 28 | Feces | 3087 |
| Healthy Control 29 | Feces | 3281 |
| Healthy Control 29 | Sigmoid Mucosa | 3302 |
| Healthy Control 30 | Sigmoid Mucosa | 253 |
| Healthy Control 30 | Feces | 2747 |
| Healthy Control 31 | Sigmoid Mucosa | 856 |
| Healthy Control 31 | Feces | 5256 |
| Healthy Control 32 | Sigmoid Mucosa | 760 |
| Healthy Control 32 | Feces | 1037 |
| Healthy Control 33 | Sigmoid Mucosa | 5062 |
| Healthy Control 34 | Feces | 3182 |
| Healthy Control 34 | Sigmoid Mucosa | 3907 |
| Healthy Control 35 | Sigmoid Mucosa | 1131 |
| Healthy Control 35 | Feces | 2911 |
| Healthy Control 36 | Sigmoid Mucosa | 647 |
| Healthy Control 37 | Sigmoid Mucosa | 2848 |
| Healthy Control 37 | Feces | 3754 |
| Healthy Control 38 | Sigmoid Mucosa | 642 |
| Healthy Control 38 | Feces | 3853 |
| Healthy Control 39 | Sigmoid Mucosa | 1731 |
| Healthy Control 39 | Feces | 3350 |
| Healthy Control 40 | Sigmoid Mucosa | 455 |
| Healthy Control 40 | Feces | 2492 |
| Healthy Control 41 | Sigmoid Mucosa | 373 |
| Healthy Control 41 | Feces | 2157 |
| Healthy Control 42 | Sigmoid Mucosa | 365 |
| Healthy Control 43 | Sigmoid Mucosa | 1263 |
| Healthy Control 43 | Feces | 4334 |
| Healthy Control 44 | Sigmoid Mucosa | 393 |
| Healthy Control 44 | Feces | 2801 |
