# Supplementary material for: Lower Neighborhood Socioeconomic Status Associated with Reduced Diversity of the Colonic Microbiota in Healthy Adults
Source: PLoS One. 2016 Feb 9;11(2):e0148952. doi: 10.1371/journal.pone.0148952 (PMC4747579; doi:10.1371/journal.pone.0148952)
Supplement: S2 Table — The number of OTUs calculated from RDP10 tables for each taxonomic resolution (Phylum to Genus) with QIIME analysis. Healthy control subjects (N = 44). A total of N = 67 samples: N = 41 sigmoid, N = 26 feces. (DOCX) [file pone.0148952.s002.docx]

**S2 Table. Summary of the numbers of OTUs across healthy control subject’s endoscopic specimen samples at each taxonomic resolution.**

|  | | **Number of OTUs** | **Number of OTUs** | **Number of OTUs** | **Number of OTUs** | **Number of OTUs** |
| --- | --- | --- | --- | --- | --- | --- |
| **Study Codes** | **Endoscopic Sample Type** | **Phylum** | **Class** | **Order** | **Family** | **Genus** |
| Healthy Control 1 | Sigmoid Mucosa | 6 | 9 | 10 | 19 | 37 |
| Healthy Control 2 | Sigmoid Mucosa | 7 | 11 | 15 | 33 | 52 |
| Healthy Control 3 | Sigmoid Mucosa | 6 | 8 | 8 | 19 | 30 |
| Healthy Control 4 | Sigmoid Mucosa | 8 | 12 | 15 | 31 | 48 |
| Healthy Control 5 | Sigmoid Mucosa | 6 | 13 | 14 | 22 | 39 |
| Healthy Control 6 | Sigmoid Mucosa | 6 | 9 | 12 | 29 | 50 |
| Healthy Control 7 | Sigmoid Mucosa | 6 | 11 | 12 | 24 | 40 |
| Healthy Control 8 | Sigmoid Mucosa | 6 | 11 | 12 | 25 | 38 |
| Healthy Control 9 | Sigmoid Mucosa | 8 | 19 | 23 | 46 | 72 |
| Healthy Control 10 | Sigmoid Mucosa | 19 | 32 | 56 | 95 | 129 |
| Healthy Control 11 | Sigmoid Mucosa | 8 | 14 | 16 | 34 | 53 |
| Healthy Control 12 | Sigmoid Mucosa | 6 | 13 | 14 | 30 | 53 |
| Healthy Control 13 | Sigmoid Mucosa | 6 | 13 | 17 | 36 | 53 |
| Healthy Control 14 | Sigmoid Mucosa | 7 | 13 | 16 | 30 | 50 |
| Healthy Control 15 | Sigmoid Mucosa | 5 | 11 | 16 | 29 | 45 |
| Healthy Control 16 | Feces | 5 | 7 | 7 | 17 | 29 |
| Healthy Control 17 | Feces | 6 | 9 | 10 | 18 | 40 |
| Healthy Control 17 | Sigmoid Mucosa | 6 | 8 | 8 | 24 | 42 |
| Healthy Control 18 | Feces | 5 | 8 | 9 | 16 | 30 |
| Healthy Control 18 | Sigmoid Mucosa | 8 | 15 | 21 | 39 | 57 |
| Healthy Control 19 | Feces | 4 | 7 | 9 | 16 | 27 |
| Healthy Control 20 | Feces | 4 | 7 | 8 | 16 | 31 |
| Healthy Control 20 | Sigmoid Mucosa | 6 | 11 | 11 | 26 | 39 |
| Healthy Control 21 | Feces | 5 | 8 | 8 | 21 | 37 |
| Healthy Control 21 | Sigmoid Mucosa | 8 | 14 | 15 | 20 | 26 |
| Healthy Control 22 | Feces | 6 | 8 | 9 | 15 | 25 |
| Healthy Control 22 | Sigmoid Mucosa | 7 | 13 | 17 | 37 | 56 |
| Healthy Control 23 | Feces | 4 | 6 | 7 | 13 | 31 |
| Healthy Control 23 | Sigmoid Mucosa | 8 | 12 | 12 | 24 | 40 |
| Healthy Control 24 | Feces | 5 | 7 | 7 | 18 | 33 |
| Healthy Control 24 | Sigmoid Mucosa | 6 | 11 | 13 | 29 | 51 |
| Healthy Control 25 | Feces | 3 | 6 | 7 | 15 | 33 |
| Healthy Control 25 | Sigmoid Mucosa | 5 | 8 | 10 | 26 | 44 |
| Healthy Control 26 | Feces | 4 | 7 | 8 | 15 | 33 |
| Healthy Control 26 | Sigmoid Mucosa | 5 | 10 | 11 | 21 | 30 |
| Healthy Control 27 | Feces | 5 | 7 | 7 | 18 | 30 |
| Healthy Control 28 | Feces | 5 | 9 | 10 | 22 | 41 |
| Healthy Control 28 | Sigmoid Mucosa | 6 | 10 | 12 | 23 | 34 |
| Healthy Control 29 | Feces | 7 | 10 | 10 | 24 | 40 |
| Healthy Control 29 | Sigmoid Mucosa | 9 | 17 | 23 | 46 | 76 |
| Healthy Control 30 | Feces | 5 | 10 | 10 | 23 | 43 |
| Healthy Control 30 | Sigmoid Mucosa | 5 | 11 | 15 | 27 | 37 |
| Healthy Control 31 | Feces | 5 | 8 | 9 | 19 | 36 |
| Healthy Control 31 | Sigmoid Mucosa | 6 | 11 | 13 | 27 | 46 |
| Healthy Control 32 | Feces | 5 | 7 | 8 | 20 | 36 |
| Healthy Control 32 | Sigmoid Mucosa | 8 | 14 | 18 | 39 | 57 |
| Healthy Control 33 | Sigmoid Mucosa | 9 | 17 | 27 | 49 | 76 |
| Healthy Control 34 | Feces | 6 | 9 | 11 | 26 | 47 |
| Healthy Control 34 | Sigmoid Mucosa | 8 | 19 | 30 | 57 | 96 |
| Healthy Control 35 | Feces | 4 | 6 | 7 | 16 | 31 |
| Healthy Control 35 | Sigmoid Mucosa | 6 | 14 | 20 | 40 | 64 |
| Healthy Control 36 | Sigmoid Mucosa | 6 | 12 | 13 | 26 | 39 |
| Healthy Control 37 | Feces | 6 | 9 | 9 | 20 | 40 |
| Healthy Control 37 | Sigmoid Mucosa | 8 | 21 | 35 | 67 | 110 |
| Healthy Control 38 | Feces | 7 | 11 | 12 | 26 | 45 |
| Healthy Control 38 | Sigmoid Mucosa | 6 | 12 | 13 | 24 | 40 |
| Healthy Control 39 | Feces | 4 | 6 | 6 | 16 | 28 |
| Healthy Control 39 | Sigmoid Mucosa | 7 | 15 | 25 | 49 | 78 |
| Healthy Control 40 | Feces | 5 | 9 | 10 | 21 | 39 |
| Healthy Control 40 | Sigmoid Mucosa | 5 | 9 | 9 | 20 | 30 |
| Healthy Control 41 | Feces | 5 | 9 | 10 | 22 | 40 |
| Healthy Control 41 | Sigmoid Mucosa | 6 | 12 | 13 | 24 | 35 |
| Healthy Control 42 | Sigmoid Mucosa | 6 | 10 | 11 | 23 | 36 |
| Healthy Control 42 | Feces | 5 | 7 | 7 | 18 | 36 |
| Healthy Control 43 | Sigmoid Mucosa | 8 | 17 | 28 | 48 | 69 |
| Healthy Control 44 | Feces | 5 | 8 | 9 | 16 | 33 |
| Healthy Control 44 | Sigmoid Mucosa | 6 | 11 | 12 | 23 | 36 |
