# Supplementary material for: Lower Neighborhood Socioeconomic Status Associated with Reduced Diversity of the Colonic Microbiota in Healthy Adults
Source: PLoS One. 2016 Feb 9;11(2):e0148952. doi: 10.1371/journal.pone.0148952 (PMC4747579; doi:10.1371/journal.pone.0148952)
Supplement: S3 Table — The alpha-diversity statistics and observed species numbers from QIIME sequence clusters. Healthy control subjects (N = 44). A total of N = 67 samples: N = 41 sigmoid, N = 26 feces. (DOCX) [file pone.0148952.s003.docx]

**S3 Table. Alpha-diversity statistics and observed species numbers from QIIME Alpha-diversity.py for healthy control subject’s endoscopic specimen samples**

| **Sample ID** | **Endoscopic Sample Type** | **ACE** | **Simpson** | **chao1** | **chao1 lower bound** | **chao1 upper bound** | **Shannon** | **PD Whole tree** | **Observed species** | **Reads** |
| --- | --- | --- | --- | --- | --- | --- | --- | --- | --- | --- |
| Healthy Control 1 | Sigmoid Mucosa | 1559.1 | 1.0 | 1415.4 | 1114.8 | 1847.8 | 6.5 | 25.2 | 429 | 1629 |
| Healthy Control 2 | Sigmoid Mucosa | 1482.2 | 1.0 | 1414.0 | 1133.9 | 1811.0 | 7.1 | 26.2 | 463 | 1588 |
| Healthy Control 3 | Sigmoid Mucosa | 617.2 | 0.9 | 550.9 | 475.3 | 663.9 | 5.6 | 21.1 | 322 | 2023 |
| Healthy Control 4 | Sigmoid Mucosa | 1827.0 | 0.9 | 1717.4 | 1434.6 | 2096.8 | 6.4 | 32.2 | 607 | 2482 |
| Healthy Control 5 | Sigmoid Mucosa | 528.5 | 0.9 | 453.1 | 369.9 | 584.9 | 5.0 | 16.7 | 228 | 1305 |
| Healthy Control 6 | Sigmoid Mucosa | 1322.3 | 1.0 | 1258.7 | 1019.5 | 1596.6 | 7.3 | 31.1 | 440 | 1184 |
| Healthy Control 7 | Sigmoid Mucosa | 2527.9 | 0.9 | 2140.9 | 1816.4 | 2564.0 | 6.7 | 39.1 | 748 | 3371 |
| Healthy Control 8 | Sigmoid Mucosa | 513.6 | 1.0 | 486.3 | 391.0 | 639.5 | 6.3 | 16.8 | 235 | 1037 |
| Healthy Control 9 | Sigmoid Mucosa | 1170.3 | 0.9 | 1133.2 | 973.4 | 1353.7 | 6.2 | 35.1 | 554 | 4276 |
| Healthy Control 10 | Sigmoid Mucosa | 1257.8 | 1.0 | 1226.4 | 1002.2 | 1542.4 | 7.5 | 37.6 | 456 | 1269 |
| Healthy Control 11 | Sigmoid Mucosa | 566.4 | 1.0 | 547.5 | 455.5 | 689.9 | 6.5 | 23.6 | 288 | 1187 |
| Healthy Control 12 | Sigmoid Mucosa | 1816.7 | 1.0 | 1688.0 | 1386.2 | 2100.1 | 7.4 | 38.5 | 561 | 1805 |
| Healthy Control 13 | Sigmoid Mucosa | 2059.2 | 1.0 | 1920.8 | 1558.7 | 2415.7 | 7.3 | 35.7 | 572 | 1762 |
| Healthy Control 14 | Sigmoid Mucosa | 1461.9 | 1.0 | 1373.0 | 1097.7 | 1764.6 | 6.7 | 29.1 | 446 | 1720 |
| Healthy Control 15 | Sigmoid Mucosa | 1420.0 | 1.0 | 1270.1 | 1029.4 | 1609.9 | 6.6 | 25.1 | 446 | 1753 |
| Healthy Control 16 | Feces | 2469.5 | 1.0 | 2140.3 | 1877.1 | 2473.5 | 7.8 | 37.3 | 887 | 2850 |
| Healthy Control 17 | Feces | 3706.2 | 1.0 | 2961.6 | 2468.2 | 3601.3 | 8.1 | 37.4 | 805 | 1908 |
| Healthy Control 17 | Sigmoid Mucosa | 1940.3 | 0.9 | 1668.9 | 1408.5 | 2015.0 | 6.4 | 34.1 | 618 | 2070 |
| Healthy Control 18 | Feces | 1801.4 | 1.0 | 1645.3 | 1397.3 | 1974.0 | 7.0 | 28.7 | 635 | 2296 |
| Healthy Control 18 | Sigmoid Mucosa | 1376.7 | 1.0 | 1350.6 | 1101.5 | 1702.0 | 6.9 | 29.3 | 494 | 2539 |
| Healthy Control 19 | Feces | 2099.8 | 1.0 | 1917.1 | 1618.2 | 2311.3 | 7.5 | 30.3 | 680 | 2194 |
| Healthy Control 20 | Feces | 3306.3 | 1.0 | 2937.7 | 2516.2 | 3471.0 | 8.1 | 41.0 | 928 | 2329 |
| Healthy Control 20 | Sigmoid Mucosa | 1451.8 | 1.0 | 1316.1 | 1043.9 | 1706.8 | 7.1 | 25.2 | 419 | 1251 |
| Healthy Control 21 | Feces | 3321.6 | 1.0 | 3008.6 | 2576.0 | 3556.4 | 7.8 | 44.1 | 953 | 2749 |
| Healthy Control 21 | Sigmoid Mucosa | 132.0 | 1.0 | 124.3 | 98.6 | 181.9 | 5.5 | 7.3 | 78 | 233 |
| Healthy Control 22 | Feces | 491.8 | 1.0 | 459.0 | 388.4 | 568.7 | 7.0 | 15.3 | 261 | 729 |
| Healthy Control 22 | Sigmoid Mucosa | 590.5 | 0.9 | 614.4 | 504.7 | 785.1 | 5.7 | 18.3 | 308 | 2492 |
| Healthy Control 23 | Feces | 2741.0 | 1.0 | 2512.6 | 2197.0 | 2908.8 | 8.2 | 42.2 | 964 | 2715 |
| Healthy Control 23 | Sigmoid Mucosa | 1414.9 | 1.0 | 1211.4 | 984.0 | 1532.7 | 7.3 | 28.8 | 433 | 1256 |
| Healthy Control 24 | Feces | 2884.3 | 1.0 | 2402.9 | 2035.2 | 2877.2 | 8.3 | 35.5 | 766 | 1548 |
| Healthy Control 24 | Sigmoid Mucosa | 2662.0 | 0.8 | 2426.3 | 2112.0 | 2824.4 | 4.3 | 43.8 | 933 | 9282 |
| Healthy Control 25 | Feces | 2909.8 | 1.0 | 2607.8 | 2249.6 | 3061.9 | 7.8 | 35.6 | 912 | 2812 |
| Healthy Control 25 | Sigmoid Mucosa | 1099.8 | 1.0 | 1364.9 | 1006.4 | 1919.5 | 6.7 | 24.1 | 351 | 1174 |
| Healthy Control 26 | Feces | 4257.2 | 1.0 | 3568.8 | 3071.0 | 4189.9 | 8.2 | 43.8 | 1062 | 2555 |
| Healthy Control 26 | Sigmoid Mucosa | 192.5 | 0.9 | 189.4 | 128.9 | 321.8 | 5.0 | 8.7 | 78 | 222 |
| Healthy Control 27 | Feces | 3589.5 | 1.0 | 2830.9 | 2433.3 | 3333.2 | 8.3 | 40.2 | 925 | 2282 |
| Healthy Control 28 | Feces | 4810.5 | 1.0 | 3920.3 | 3428.8 | 4522.0 | 8.1 | 52.3 | 1235 | 3087 |
| Healthy Control 28 | Sigmoid Mucosa | 578.1 | 0.9 | 511.5 | 371.1 | 752.8 | 5.9 | 12.7 | 176 | 527 |
| Healthy Control 29 | Feces | 4789.0 | 1.0 | 4109.2 | 3588.7 | 4746.7 | 8.3 | 55.3 | 1274 | 3281 |
| Healthy Control 29 | Sigmoid Mucosa | 2035.8 | 1.0 | 1972.9 | 1696.2 | 2333.6 | 7.7 | 45.1 | 783 | 3302 |
| Healthy Control 30 | Feces | 2167.8 | 1.0 | 1976.2 | 1717.5 | 2307.9 | 7.4 | 38.4 | 799 | 2747 |
| Healthy Control 30 | Sigmoid Mucosa | 189.3 | 1.0 | 163.0 | 124.2 | 243.6 | 5.5 | 10.5 | 88 | 253 |
| Healthy Control 31 | Feces | 6606.8 | 1.0 | 6181.6 | 5488.2 | 7006.5 | 8.5 | 75.6 | 1829 | 5256 |
| Healthy Control 31 | Sigmoid Mucosa | 1043.0 | 1.0 | 1007.6 | 768.6 | 1371.9 | 6.8 | 24.5 | 313 | 856 |
| Healthy Control 32 | Feces | 2381.3 | 1.0 | 2068.3 | 1679.1 | 2594.7 | 8.3 | 31.1 | 576 | 1037 |
| Healthy Control 32 | Sigmoid Mucosa | 605.4 | 1.0 | 556.2 | 455.5 | 711.7 | 7.1 | 23.4 | 270 | 760 |
| Healthy Control 33 | Sigmoid Mucosa | 1864.9 | 0.9 | 1763.9 | 1524.1 | 2076.8 | 6.1 | 39.7 | 739 | 5062 |
| Healthy Control 34 | Feces | 4856.2 | 1.0 | 3948.7 | 3475.5 | 4524.8 | 8.6 | 56.6 | 1299 | 3182 |
| Healthy Control 34 | Sigmoid Mucosa | 1433.5 | 1.0 | 1384.7 | 1207.7 | 1620.4 | 7.3 | 37.2 | 675 | 3907 |
| Healthy Control 35 | Feces | 3321.4 | 1.0 | 2979.2 | 2588.4 | 3467.9 | 8.1 | 44.1 | 1028 | 2911 |
| Healthy Control 35 | Sigmoid Mucosa | 546.9 | 1.0 | 591.5 | 453.9 | 817.2 | 6.0 | 18.9 | 239 | 1131 |
| Healthy Control 36 | Sigmoid Mucosa | 463.4 | 0.9 | 511.1 | 346.8 | 815.2 | 5.0 | 13.6 | 154 | 647 |
| Healthy Control 37 | Feces | 3783.5 | 1.0 | 3304.1 | 2982.2 | 3691.7 | 8.8 | 54.4 | 1406 | 3754 |
| Healthy Control 37 | Sigmoid Mucosa | 1105.0 | 1.0 | 1043.2 | 905.2 | 1233.1 | 6.9 | 33.6 | 538 | 2848 |
| Healthy Control 38 | Feces | 5815.0 | 1.0 | 4855.6 | 4240.1 | 5604.1 | 8.2 | 60.9 | 1391 | 3853 |
| Healthy Control 38 | Sigmoid Mucosa | 501.4 | 0.9 | 447.3 | 342.8 | 622.2 | 5.7 | 17.9 | 188 | 642 |
| Healthy Control 39 | Feces | 3950.8 | 0.9 | 3472.5 | 2989.6 | 4076.4 | 7.4 | 45.3 | 1063 | 3350 |
| Healthy Control 39 | Sigmoid Mucosa | 841.8 | 1.0 | 931.9 | 761.6 | 1181.5 | 7.0 | 26.3 | 396 | 1731 |
| Healthy Control 40 | Feces | 1964.5 | 1.0 | 1679.6 | 1410.7 | 2038.9 | 6.9 | 31.3 | 612 | 2492 |
| Healthy Control 40 | Sigmoid Mucosa | 319.8 | 0.9 | 291.0 | 217.4 | 426.0 | 5.0 | 11.6 | 129 | 455 |
| Healthy Control 41 | Feces | 2187.0 | 0.9 | 1920.1 | 1616.0 | 2321.4 | 7.0 | 31.6 | 664 | 2157 |
| Healthy Control 41 | Sigmoid Mucosa | 335.8 | 1.0 | 337.0 | 250.1 | 493.9 | 6.1 | 14.4 | 142 | 373 |
| Healthy Control 42 | Sigmoid Mucosa | 471.4 | 0.9 | 348.3 | 248.9 | 529.6 | 5.5 | 13.7 | 128 | 365 |
| Healthy Control 43 | Feces | 3347.8 | 1.0 | 2959.3 | 2662.7 | 3319.7 | 7.9 | 50.8 | 1285 | 4334 |
| Healthy Control 43 | Sigmoid Mucosa | 309.8 | 0.9 | 301.0 | 254.0 | 383.1 | 5.5 | 14.4 | 191 | 1263 |
| Healthy Control 44 | Feces | 1509.8 | 0.9 | 1511.5 | 1257.0 | 1858.4 | 5.7 | 29.1 | 556 | 2801 |
| Healthy Control 44 | Sigmoid Mucosa | 537.4 | 1.0 | 380.0 | 290.5 | 531.8 | 6.4 | 14.9 | 162 | 393 |
