# Supplementary material for: Lower Neighborhood Socioeconomic Status Associated with Reduced Diversity of the Colonic Microbiota in Healthy Adults
Source: PLoS One. 2016 Feb 9;11(2):e0148952. doi: 10.1371/journal.pone.0148952 (PMC4747579; doi:10.1371/journal.pone.0148952)
Supplement: S4 Table — Values are Pearson (for age, body mass index, alcohol) or Point-Biserial correlations (for Gender, Caucasian, African-American, and Smoking). Gender is coded as 0 = Male, 1 = Female. Caucasian, African-American, and Smoker are coded as 0 = No, 1 = Yes. For sigmoid mucosa, where n = 41, the critical value of r at α = 0.05 is 0.30. For Feces, where n = 26, the critical value of r at α = 0.05 is 0.37. * p < 0.01; ^ p < 0.001. (DOCX) [file pone.0148952.s004.docx]

**S4 Table. Correlations between neighborhoods SES, alpha-diversity indices, and covariates.**

|  | **Neighborhood SES Composite *n*=44** | **Sigmoid Mucosa Shannon Index *n*=41** | **Sigmoid Mucosa Chao1 Index *n*=41** | **Feces Shannon Index *n*=26** | **Feces Chao1 Index *n*=25** |
| --- | --- | --- | --- | --- | --- |
| Age | 0.15 | -0.06 | -0.07 | -0.08 | 0.10 |
| Gender | 0.03 | -0.05 | -0.05 | -0.14 | -0.31 |
| Caucasian | 0.47 ^ | 0.24 | 0.24 | 0.14 | 0.12 |
| African-American | -0.51 ^ | -0.21 | -0.19 | -0.01 | 0.04 |
| Body Mass Index | -0.39**^*^** | -0.27 | -0.26 | -0.24 | -0.12 |
| Current Smoker | 0.04 | 0.16 | 0.17 | -0.23 | -0.26 |
| Alcohol Use | -0.04 | 0.07 | 0.06 | 0.04 | 0.34 |

Values are Pearson (for age, body mass index, alcohol) or Point-Biserial correlations (for Gender, Caucasian, African-American, and Smoking). Gender is coded as 0 = Male, 1 = Female. Caucasian, African-American, and Smoker are coded as 0 = No, 1 = Yes. For sigmoid mucosa, where *n* = 41, the critical value of *r* at α = 0.05 is 0.30. For Feces, where *n* = 26, the critical value of *r* at α = 0.05 is 0.37. * *p* < 0.01; ^^^ *p* < 0.001.
