# Supplementary material for: Lower Neighborhood Socioeconomic Status Associated with Reduced Diversity of the Colonic Microbiota in Healthy Adults
Source: PLoS One. 2016 Feb 9;11(2):e0148952. doi: 10.1371/journal.pone.0148952 (PMC4747579; doi:10.1371/journal.pone.0148952)
Supplement: S5 Table — Table shows R2 values, reflecting percentage of variance in alpha diversity indices explained by each covariate (for age, body mass index, alcohol). Gender is coded as (0 = Male), (1 = Female). Caucasian, African-American, and Smoker are coded as (0 = No), (1 = Yes). (DOCX) [file pone.0148952.s005.docx]

**S5 Table. Percent variance in alpha diversity explained by covariates.**

|  | **Sigmoid Mucosa Shannon Index *n*=41** | **Sigmoid Mucosa Chao1 Index *n*=41** | **Feces Shannon Index *n*=26** | **Feces Chao1 Index *n*=25** |
| --- | --- | --- | --- | --- |
| Age | 0.004 | 0.005 | 0.006 | 0.010 |
| Gender | 0.003 | 0.003 | 0.020 | 0.096 |
| Caucasian | 0.058 | 0.058 | 0.020 | 0.014 |
| African-American | 0.044 | 0.036 | 0.000 | 0.002 |
| Body Mass Index | 0.073 | 0.068 | 0.058 | 0.014 |
| Current Smoker | 0.026 | 0.029 | 0.053 | 0.068 |
| Alcohol Use | 0.005 | 0.004 | 0.002 | 0.116 |

Table shows R^2^ values, reflecting percentage of variance in alpha diversity indices explained by each covariate (for age, body mass index, alcohol). Gender is coded as 0 = Male, 1 = Female. Caucasian, African-American, and Smoker are coded as 0 = No, 1 = Yes. For sigmoid mucosa, where *n* = 41, the critical value of *r* at α = 0.05 is 0.30. For Feces, where *n* = 26, the critical value of *r* at α = 0.05 is 0.37. * *p* < 0.01; ^^^ *p* < 0.001.
