# Supplementary figures and images for: Lower Neighborhood Socioeconomic Status Associated with Reduced Diversity of the Colonic Microbiota in Healthy Adults
Source: PLoS One. 2016 Feb 9;11(2):e0148952. doi: 10.1371/journal.pone.0148952 (PMC4747579; doi:10.1371/journal.pone.0148952)

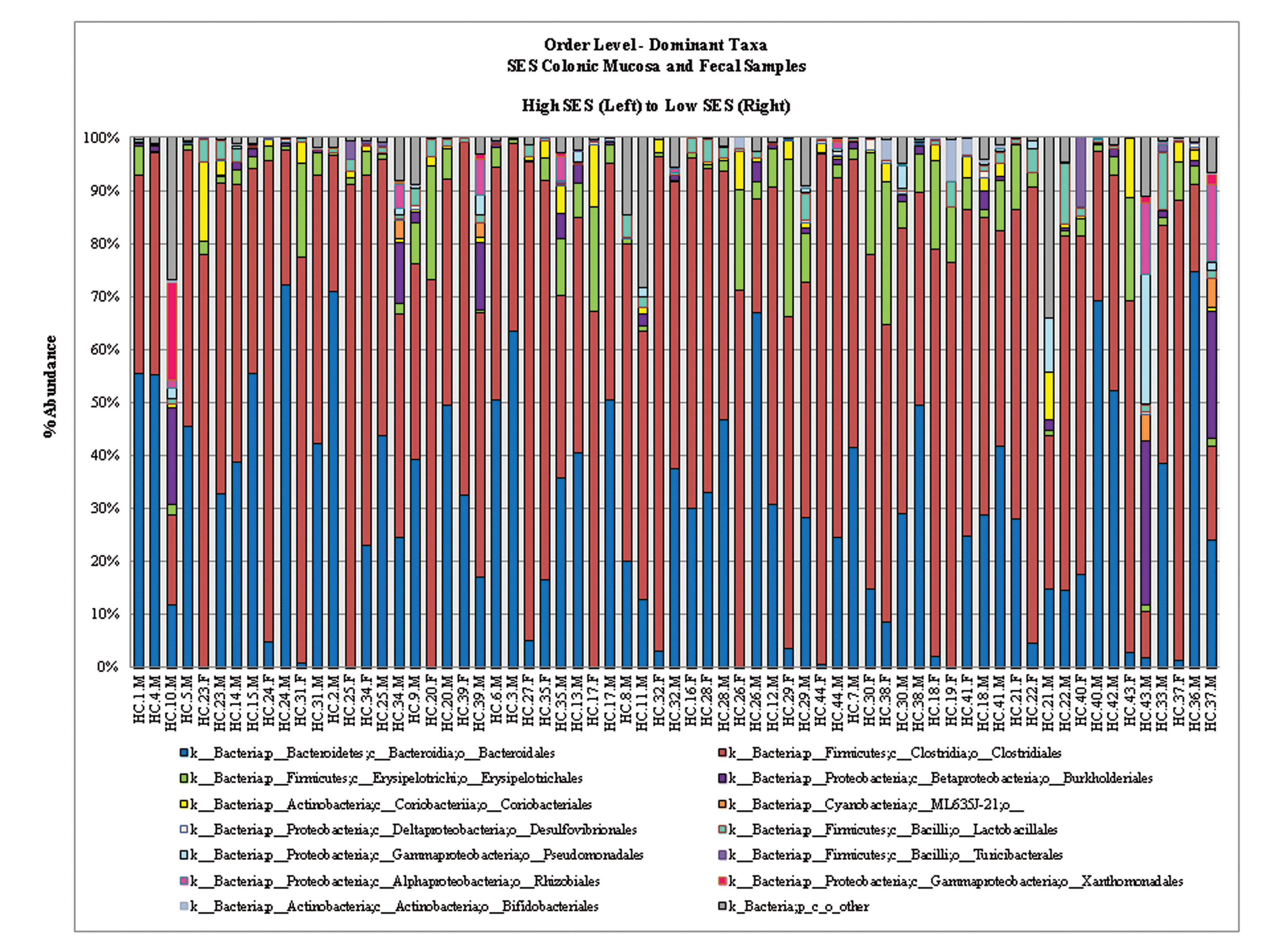

Supplement: S1 Fig — The QIIME taxa dominant abundances are presented as stacked histograms. Healthy control endoscopic specimen samples were sorted by the SES values from positive (left side of graph) to negative (right side of graph). The Bacteroidales is highlighted in blue. (TIF) [file pone.0148952.s006.tif]

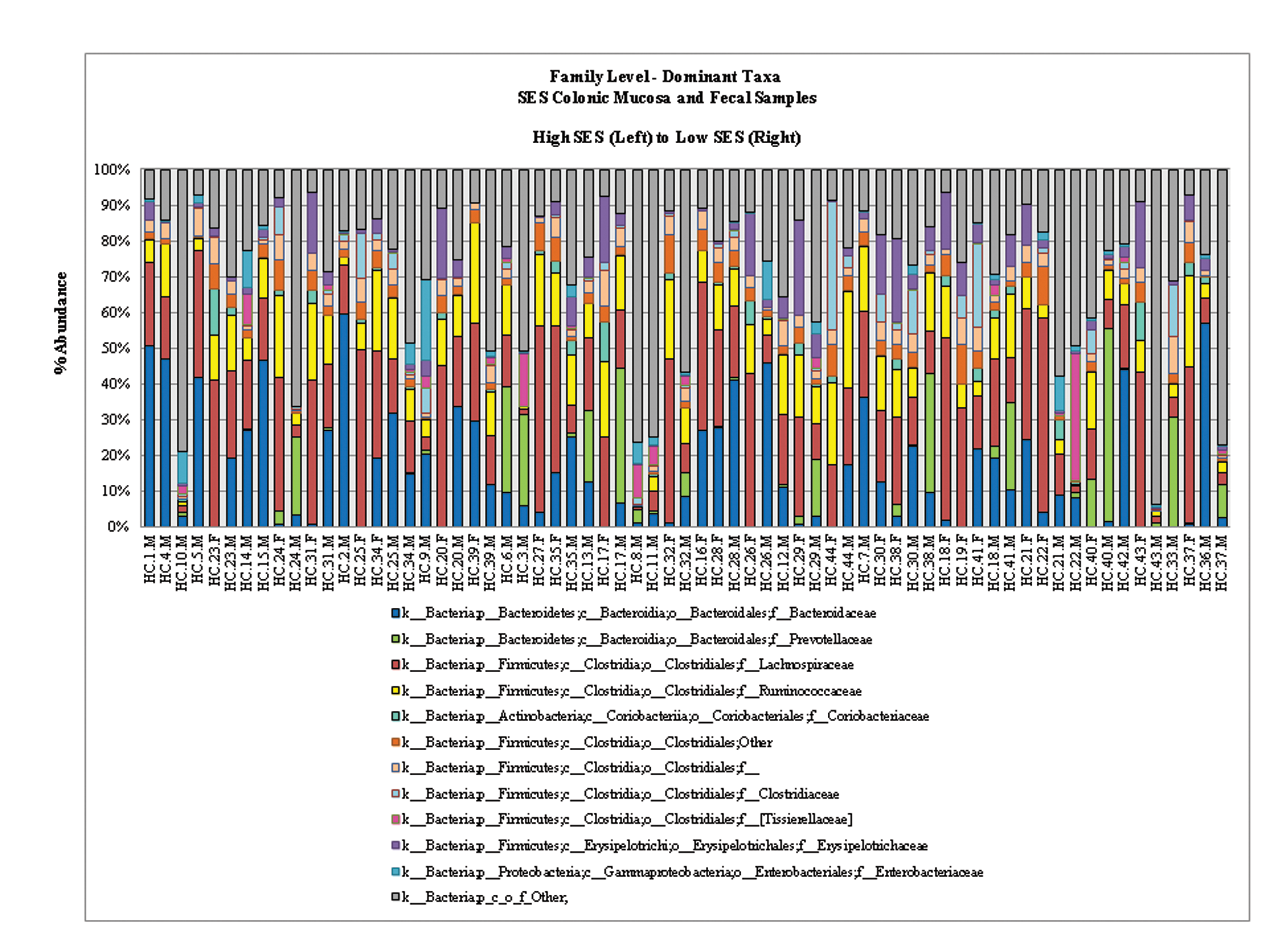

Supplement: S2 Fig — The QIIME taxa dominant abundances are presented as stacked histograms. Healthy control endoscopic specimen samples were sorted by the SES values from positive (left side of graph) to negative (right side of graph). The Bacteroidaceae is highlighted in blue. (TIF) [file pone.0148952.s007.tif]

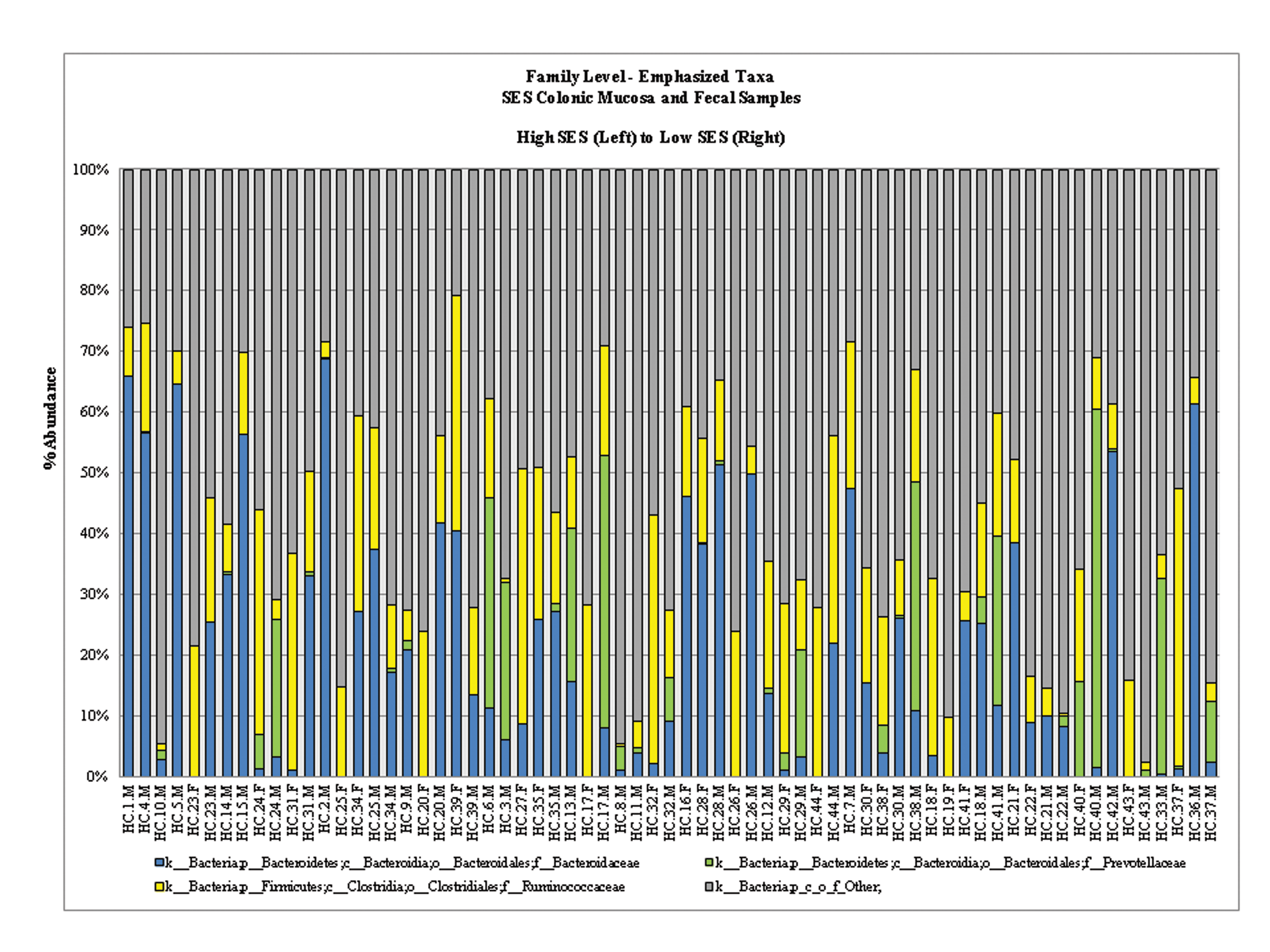

Supplement: S3 Fig — The QIIME taxa abundances are presented as stacked histograms. Healthy control endoscopic specimen samples were sorted by the SES values from positive (left side of graph) to negative (right side of graph). The Bacteroidaceae (blue), Prevotellaceae (green), and Ruminococcaceae (yellow) are highlighted. (TIF) [file pone.0148952.s008.tif]

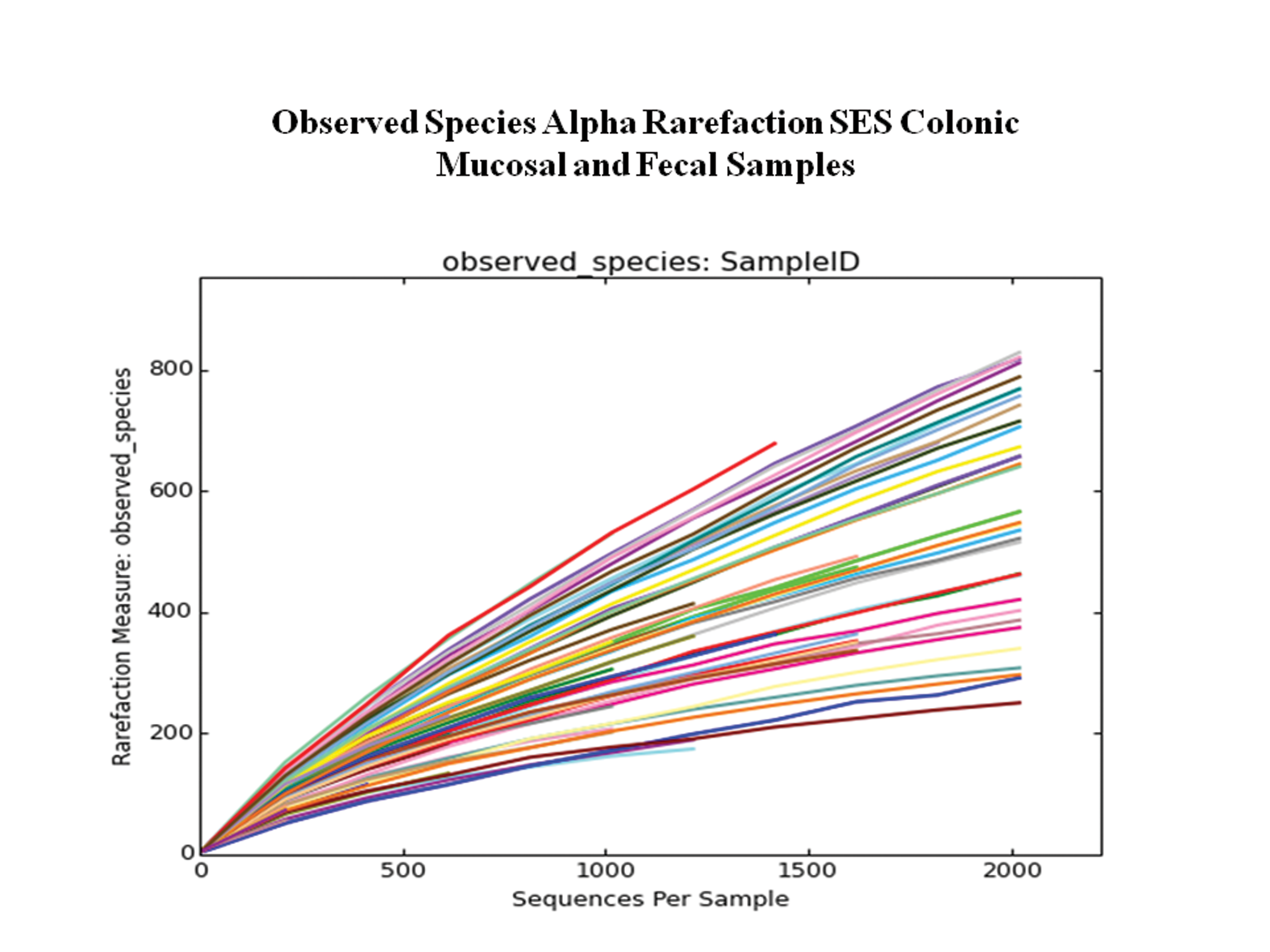

Supplement: S4 Fig — Sample rarefaction using Alpha_Diversity.py was run on the data and the sequences per healthy control endoscopic specimen sample was plotted. The rarefaction curves are done with OTUs from QIIME. The rarefaction analysis indicates that the community for most of the endoscopic specimen samples was close to being saturated. (TIF) [file pone.0148952.s009.tif]

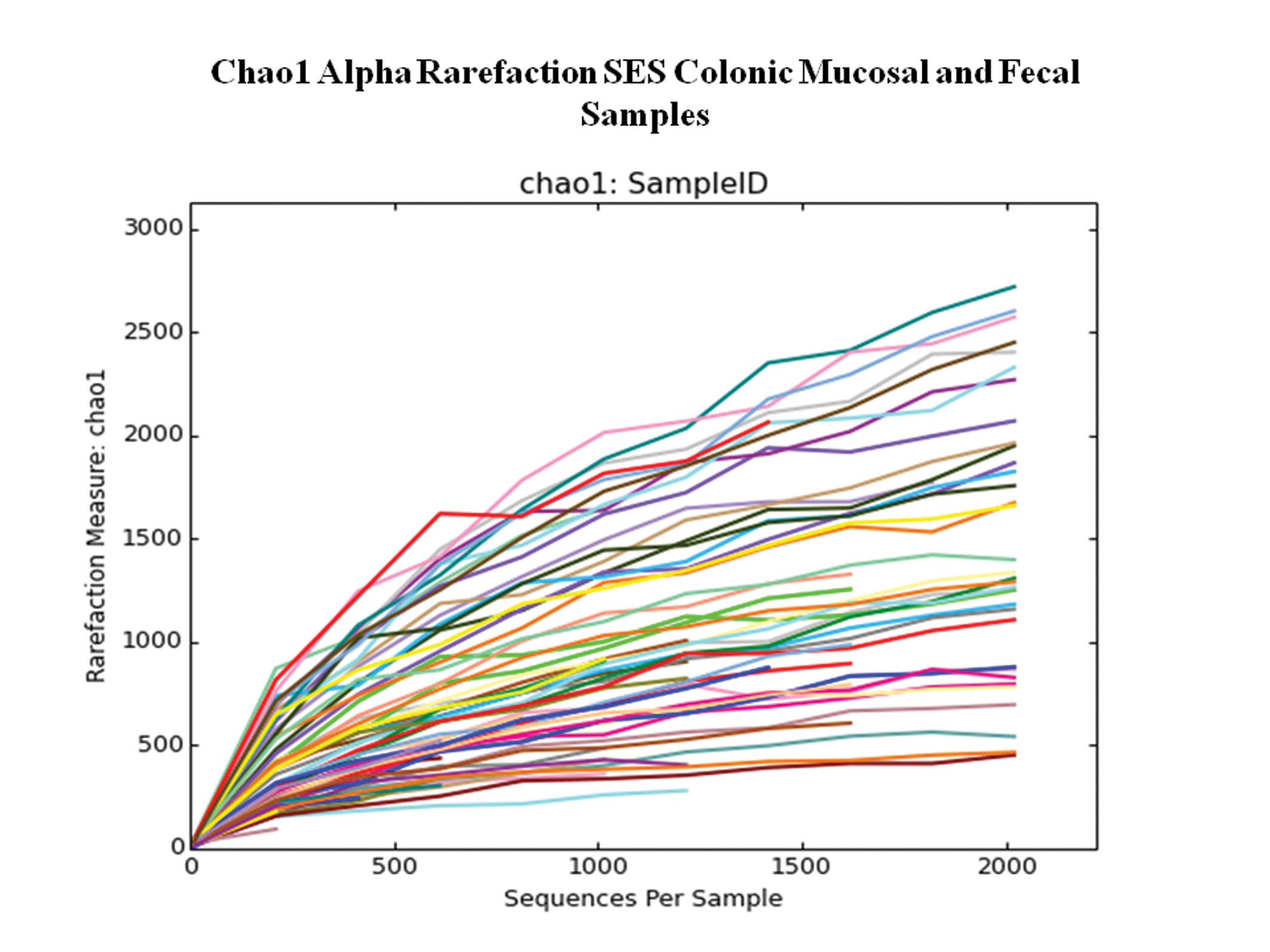

Supplement: S5 Fig — Sample rarefaction using Alpha_Diversity.py was run on the data and the sequences per healthy control endoscopic specimen sample was plotted. The rarefaction curves are done with OTUs from QIIME. The rarefaction analysis indicates that the community for most of the endoscopic specimen samples was close to being saturated. (TIF) [file pone.0148952.s010.tif]

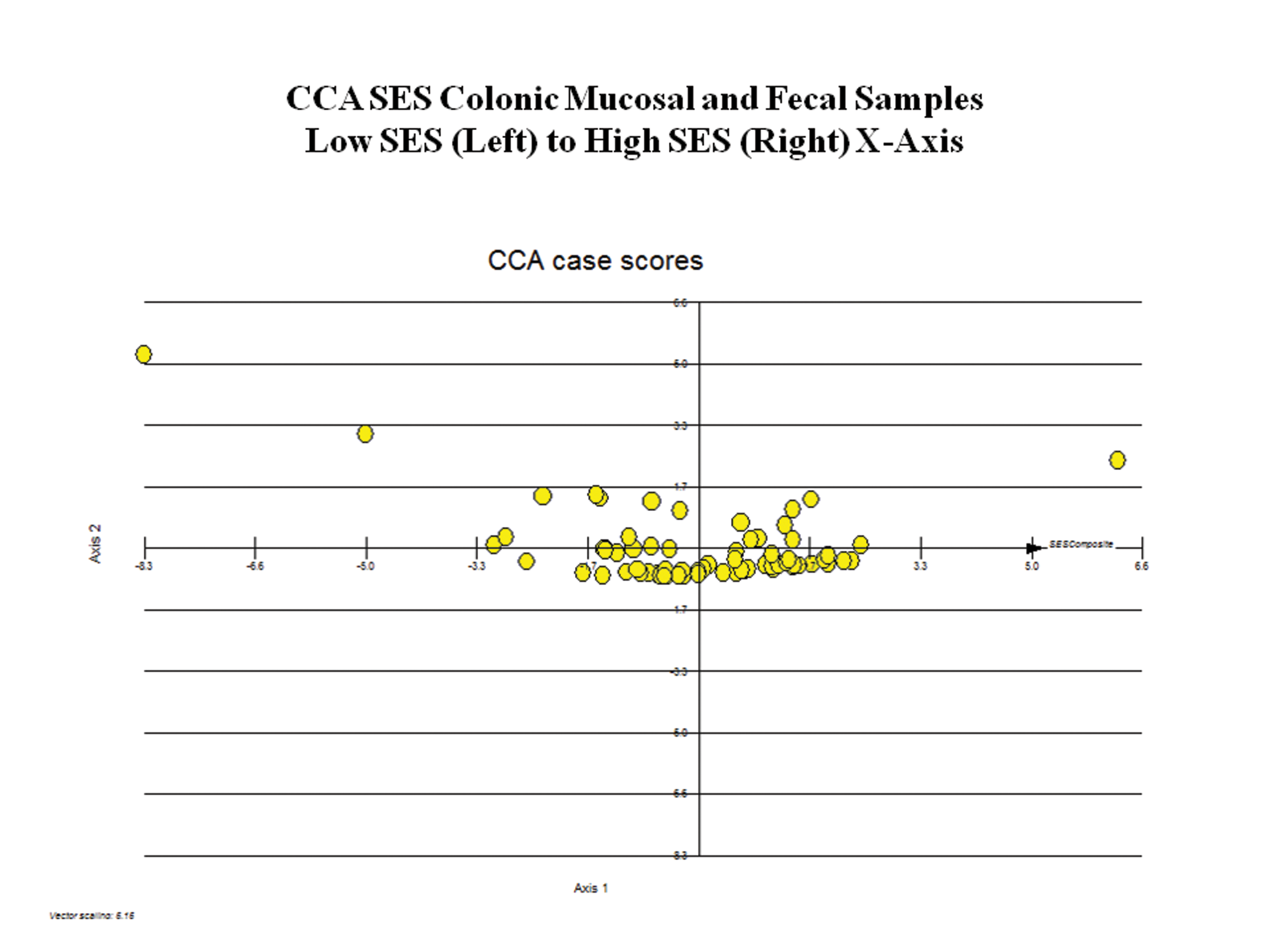

Supplement: S6 Fig — Canonical Correspondence Analysis (CCA) plot of all healthy control endoscopic specimen samples using SES as the environmental variable. SES composite CCA case scores indicated on X-axis (low SES is left; high SES high is right). (TIF) [file pone.0148952.s011.tif]

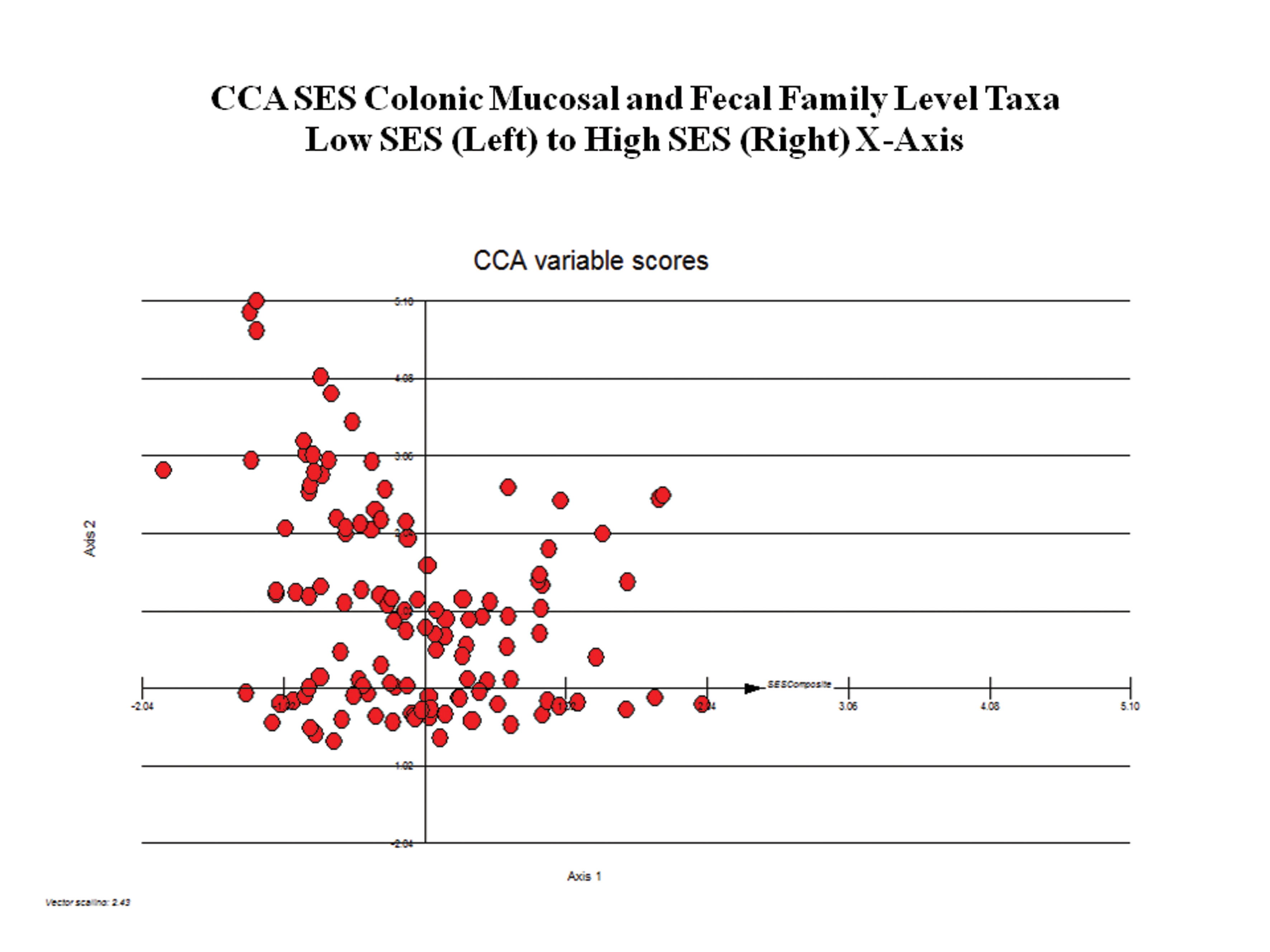

Supplement: S7 Fig — Canonical Correspondence Analysis (CCA) plot of all healthy control endoscopic specimens Family level taxa using SES as the environmental variable. SES composite CCA case scores indicated on X-axis (low SES is left; high SES high is right). (TIF) [file pone.0148952.s012.tif]

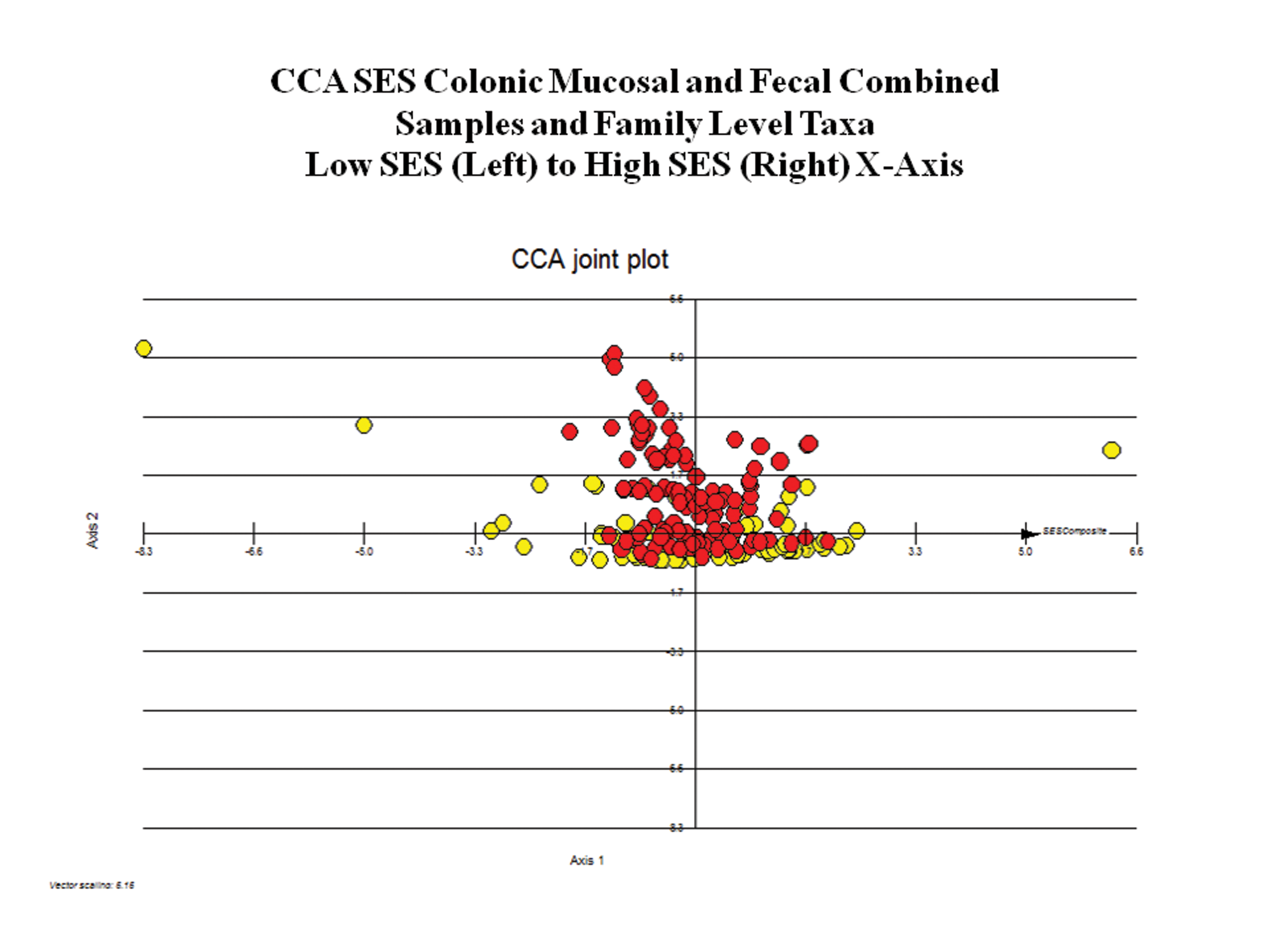

Supplement: S8 Fig — Canonical Correspondence Analysis (CCA) bi-plot of all healthy control endoscopic specimen samples and Family level taxa using SES as the environmental variable. SES composite CCA case scores indicated on X-axis (low SES is left; high SES high is right). (TIF) [file pone.0148952.s013.tif]
